# Real‐Time Monitoring of Volatile Organic Compound‐Mediated Plant Intercommunication Using Surface‐Enhanced Raman Scattering Nanosensor

**DOI:** 10.1002/advs.202412732

**Published:** 2024-12-24

**Authors:** Yun Sik Choi, Won Ki Son, Hyuna Kwak, Jiyeun Park, Sumin Choi, Daeseob Sim, Min Gyeong Kim, Hyungsuk Kimm, Hokyoung Son, Dae Hong Jeong, Seon‐Yeong Kwak

**Affiliations:** ^1^ Department of Chemistry Education College of Education Seoul National University Seoul 08826 Republic of Korea; ^2^ Department of Agriculture Forestry and Bioresources Seoul National University Seoul 08826 Republic of Korea; ^3^ Department of Agricultural Biotechnology Seoul National University Seoul 08826 Republic of Korea; ^4^ Department of Plant Science, College of Agriculture and Life Sciences Seoul National University Seoul 08826 Republic of Korea; ^5^ Research Institute of Agriculture and Life Science Seoul National University Seoul 08826 Republic of Korea; ^6^ Science Educational Research Center Seoul National University Seoul 08826 Republic of Korea

**Keywords:** interplant communication, real‐time monitoring, surface‐enhanced Raman scattering nanosensor, volatile organic compounds detection

## Abstract

Plants communicate through volatile organic compounds (VOCs), but real‐time monitoring of VOCs for plant intercommunication is not practically possible yet. A nanobionic VOC sensor plant is created to study VOC‐mediated plant intercommunication by incorporating surface‐enhanced Raman scattering (SERS) nanosensors into a living plant. This sensor allows real‐time monitoring of VOC with a sensitivity down to the parts per trillion level. A quantitative VOC diffusion model in plants is proposed to describe this extreme sensitivity. The sensor plant is paired with a customized portable Raman device, demonstrating its ability to detect multiple VOCs on‐field. The sensor demonstrated that plants collect VOCs emitted from neighboring plants and hazardous volatile chemicals in the air at a certain distance. As a feasibility study, this nanobionic VOC sensor plant successfully monitored the early stages of fungal infection in strawberry fruits. This result suggests that interfacing nanosensors with plants offers an innovative approach to studying interplant communication and can be used as a compelling tool for monitoring VOC occurrence.

## Graphical abstract

Living plants are transformed into autonomous sensors for volatile organic compounds (VOCs) by incorporating surface‐enhanced Raman scattering (SERS) nanosensors. This nanobionic sensor allows the plant to monitor VOCs in real‐time, exploiting the plant's inherent ability to communicate. Additionally, it serves as a sensitive optical sensor for VOCs, making it suitable for a wide range of applications under ambient conditions.

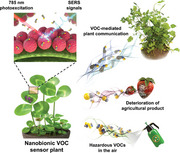

## 1 Introduction

Plants constantly perceive information from their surroundings including neighboring plants. When the first “talking tree” was introduced to the scientific community,^[^ ^1^ ^]^ followed by the reporting of scientific evidence for communication between plants,^[^ ^2^ ^]^ ideas on communication between plants were questionable and severely criticized. However, it is now generally accepted that plants emit signals under certain cues, and other plants recognize these signals and specifically react;^[^ ^3^, ^4^, ^5^ ^]^ these signals are mostly mediated by volatile organic compounds (VOCs).^[^ ^3^, ^6^ ^]^ Although studying the underlying mechanism connecting plant perception and response is an active research area, progress in studying interplant communications is relatively slow due to a lack of proper analytical tools and limited empirical data to substantiate the evolution of airborne signal emissions. Gas chromatography‐mass spectrometry (GC‐MS) is generally recognized as a standard method for analyzing VOCs.^[^ ^7^, ^8^ ^]^ However, complex configurations and labor‐intensive sampling processes make GC‐MS less suitable for real‐time monitoring of VOCs. GC‐MS requires additional components, such as a carrier gas and a pump, to transport the sample through the column and maintain a vacuum state. The sampling process for analyzing plant VOCs with GC‐MS involves extracting essential oils directly from the plant or using completely dried plant samples.^[^ ^9^, ^10^ ^]^ These destructive sampling methods cause damage plants during the VOC extraction process. Nanomaterial‐based sensing platforms have gained significant attention for practical VOC detection due to their advantages, low detection limits, and fast response times.^[^ ^11^, ^12^, ^13^, ^14^, ^15^, ^16^, ^17^ ^]^ These studies often struggle to detect and identify multiple VOCs simultaneously, the sensitivity of the reported VOC nanosensors is not on par with that of GC‐MS, and a VOC collection step is still required to obtain the signals. Nevertheless, scientists are continuing their efforts to better understand this plant communication, and recently, VOC sensory transduction in *Arabidopsis thaliana* leaves have been reported.^[^ ^18^ ^]^

In nature, plants uptake air through stomata on their leaf surface,^[^ ^19^ ^]^ which is crucial for photosynthesis. Once the VOCs enter the plant leaves, they diffuse into the cytosol after passing through the cell wall and the plasma membrane.^[^ ^20^ ^]^ Since plants metabolize VOCs that enter the cytosol through oxidation or reduction, the VOC concentration remains lower, enabling further VOC uptake.^[^ ^20^, ^21^ ^]^ Due to this continuous, inherent process of living plants, plants are active VOC samplers by entrapping volatile compounds in the atmosphere and accumulating the compounds within the leaves.

**Figure 1:**
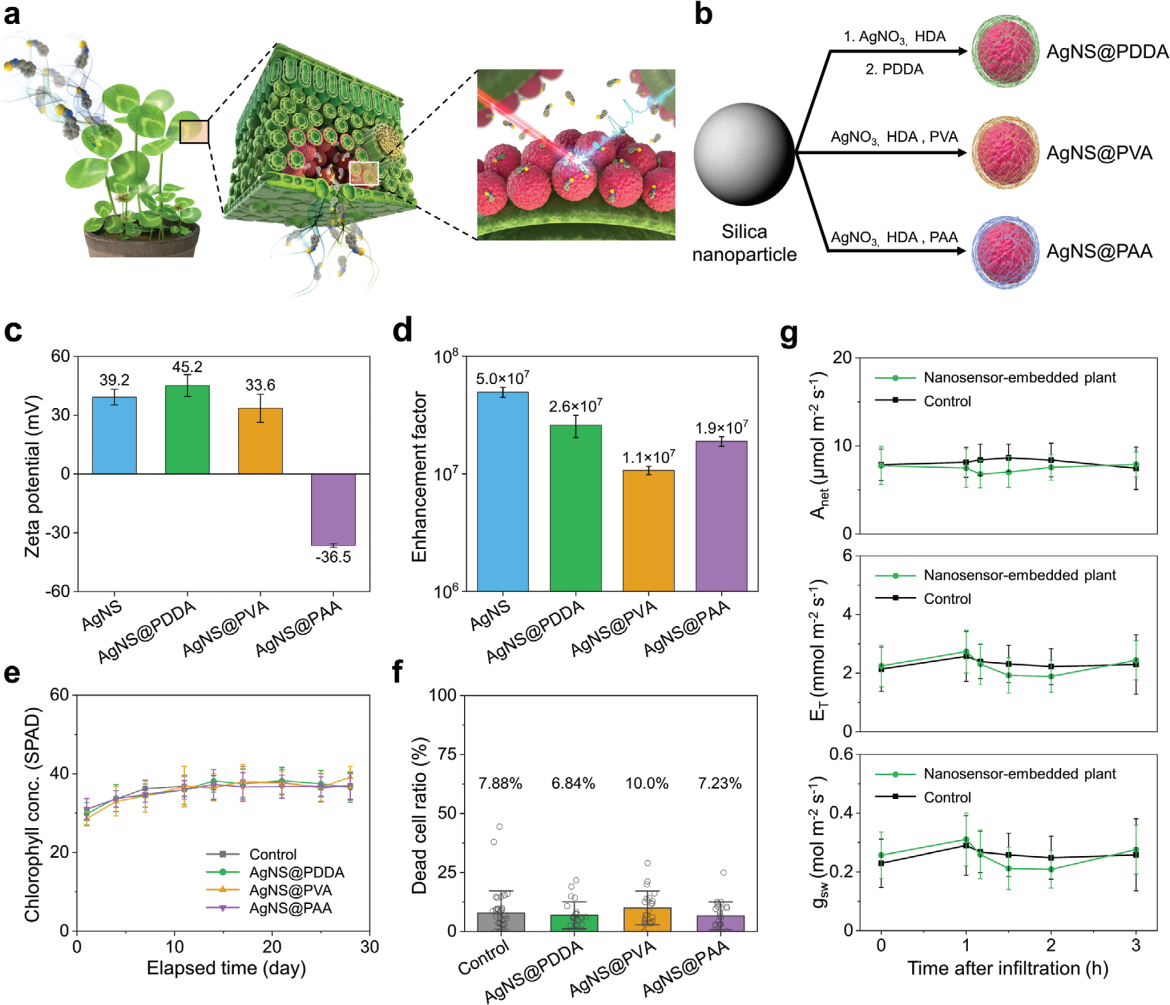
Fabrication of a nanobionic VOC sensor plant and its biocompatibility. a) Schematic of VOC sensing using a nanobionic sensor plant. b) Schematic for synthesizing polymer‐wrapped silver nanoshells (AgNS@PDDA, AgNS@PVA, and AgNS@PAA). AgNS@PDDA prepared from 3‐mercaptopropyltrimethoxysilane (MPTS)‐functionalized silica nanoparticles by reducing silver nitrate with hexadecylamine to create a bumpy silver surface that was further modified with PDDA. AgNS@PVA and AgNS@PAA were prepared similarly by modifying nanoparticle surfaces with PVA and PAA. c) Zeta potentials of AgNS@PDDA (green), AgNS@PVA (dark yellow), AgNS@PAA (purple), and bare AgNS (blue). The data are presented as mean±s.d. based on six individual experiments. d) SERS enhancement factors of AgNS@PDDA (green), AgNS@PVA (dark yellow), and AgNS@PAA (purple) compared with that of bare AgNSs (blue). SERS spectra for estimating the Raman enhancement factor were obtained with a 785 nm laser with 2 mW of incident power. e) Chlorophyll content in plants after infiltration of AgNS@PDDA (green), AgNS@PVA (dark yellow), AgNS@PAA (purple), and without nanosensors (gray) for comparison. The data are presented as mean±s.d. based on six individual experiments at each point. f) Dead cell ratios of propidium iodide‐stained leaf epidermal cell walls of clover after infiltration of AgNS@PDDA (gray), AgNS@PVA (dark yellow), AgNS@PAA (purple), and without nanosensors (gray) for comparison. The data are presented as mean±s.d. based on twenty‐four individual experiments. g) Effect of the nanoparticle infiltration on the physiological properties of the clover leaves. Net assimilation (photosynthesis – respiration, A_net_), evapotranspiration (E_T_), and stomatal conductance (g_sw_) of clover were measured with AgNS@PDDA infiltration (green) and without infiltration (black). The data are presented as mean±s.d. based on ten individual experiments.

Here, we introduce a nanobionic VOC sensor plant that utilizes the plant's inheritability to communicate using VOCs (**Figure** **1**a). Plant nanobionics, initially introduced^[^ ^22^ ^]^ and expanded upon in subsequent studies,^[^ ^23^, ^24^, ^25^, ^26^, ^27^ ^]^ refers to the engineering of living plants, plant tissues, or organelles by integrating non‐native nanomaterials. The goal is to enhance existing functions or introduce new ones in plants. Using this plant nanobionic approach, we engineer living plants to create an autonomous VOC sensor by introducing surface‐enhanced Raman scattering (SERS) nanoparticles. The plasmonic nanostructure can enhance the Raman scattering of molecules,^[^ ^28^ ^]^ which provides the potential for single‐molecule level sensitivity.^[^ ^29^, ^30^ ^]^ Additionally, SERS has spectral specificity due to its fingerprint characteristics of analyte molecules; thus, Raman molecular fingerprinting enables extensive multiple constituent detection and intuitive molecular identification. In addition, the plasmonic nanoprobe needs to be effective in the near‐infrared (NIR) region to circumvent chlorophyll autofluorescence, so silver bumpy nanoshells (AgNSs) are used.^[^ ^31^, ^32^ ^]^ Since plant nanobionics are species‐independent, a variety of plants can be engineered; in our study, we chose white clover (*Trifolium repens*), which is a widely distributed, globally cultivated herbaceous perennial plant, due to its convenience and compatibility with lawns, crops, and vegetable rows.^[^ ^33^, ^34^ ^]^ This nanobionic VOC sensor plant has the potential to study interplant communications and functions as a highly sensitive optical VOC sensor for wide‐ranging applications under ambient conditions.

## 2 Results

### 2.1 Design and Synthesis of Highly Sensitive SERS‐Active Nanoparticles for Living Plant Applications

Ag bumpy nanoshells (AgNSs) were synthesized on thiolated silica nanoparticles by reducing silver nitrate with hexadecylamine.^[^ ^32^ ^]^ As previously reported, the bumpy surface‐structured AgNSs showed a large enhancement (*ca*. 5.0 × 10^7^) in Raman scattering in the NIR window away from the chlorophyll autofluorescence region.^[^ ^31^, ^32^ ^]^ This nanostructure has been designed for label‐free detection, enabling the simultaneous identification of multiple molecules. The nanoparticle dimensions are small enough to penetrate stomatal pores, yet large enough to remain in the intercellular spaces of the leaves.^[^ ^35^, ^36^, ^37^ ^]^ This size allows AgNSs to interact directly with VOCs during the gas exchange process of the plant. The nanoparticle surface was modified with three different water‐soluble polymers, poly(diallyldimethylammonium chloride) (PDDA), poly(vinyl alcohol) (PVA), and poly(acrylic acid) (PAA), to ensure the colloidal stability of AgNSs under the in vivo aqueous conditions in living plants (Figure 1b,c). Moreover, these polymers can help attract analytes close to the silver surface by noncovalent interactions for sufficient Raman scattering enhancement. PDDA is a cationic polymer, has good biocompatibility with plant leaves, and can form electrostatic interactions and hydrogen bonds with various plant signaling molecules.^[^ ^31^ ^]^ PVA has no charged functional groups; instead, the alkyl chain backbone and hydroxyl groups can interact with the analytes that have no charged functional groups and so are not attracted to the PDDA polymer chains, most likely through hydrogen bonding with the hydroxyl groups.^[^ ^38^, ^39^ ^]^ PAA is a negatively charged polymer that attracts positively charged molecules to the nanoparticle surfaces.^[^ ^40^ ^]^ These polymers enabled AgNSs to attract target molecules near the “hotspot” sites between silver nanostructures, where strong Raman signal enhancement occurs. The resulting PDDA‐wrapped AgNSs (AgNS@PDDA), PVA‐wrapped AgNSs (AgNS@PVA) and PAA‐wrapped AgNSs (AgNS@PAA) maintained the bumpy surface structure of the AgNSs after functionalization of the AgNS surface with the polymers (Figure S1, Supporting Information). Upon polymer wrapping, the hydrodynamic diameter slightly increased from 300 nm to 315–360 nm (Figure S2, Supporting Information). Polymer‐wrapped AgNSs exhibited broad optical extinction similar to that of bare AgNS in the NIR region (Figure S3, Supporting Information), which is suitable for collecting SERS spectra of analytes under 785‐nm excitation without interference from chlorophyll fluorescence in plants. The Raman enhancement factors of AgNS@PDDA, AgNS@PVA, and AgNS@PAA were estimated to be 2.6 × 10^7^, 1.1 × 10^7^, and 1.9 × 10^7^, respectively, by using 4‐fluorobenzenthiol (4‐FBT) molecules;^[^ ^41^ ^]^ these results indicated that the nanosensors were highly sensitive for detecting trace amounts of analytes (Figure 1d). Polymer‐wrapped AgNS was introduced into the living white clover plant by infiltration through stomata on the abaxial side of the leaves using a needleless syringe (Figure S4, Supporting Information).^[^ ^26^ ^]^ The size of the stomatal aperture was ~3 µm (Figure S5, Supporting Information); this aperture size was sufficient to allow nanoparticles to enter plant leaves non‐destructively. Raman intensity maps illustrate that 99% of the infiltrated nanosensors were localized within a radius less than 5 mm from the infiltration point (Figure S6, Supporting Information). The biocompatibility of nanosensors was evaluated by monitoring the chlorophyll content in nanosensor‐embedded leaves, an indicator of leaf lifespan. The chlorophyll content of leaves infiltrated with AgNS@PDDA, AgNS@PVA, and AgNS@PAA did not significantly differ from that of leaves infiltrated with MES buffer over 4 weeks (Figure 1e); these results indicate that leaf senescence barely occurred after the introduction of the nanosensors. A propidium iodide staining test was also carried out to observe biological stresses at the cellular level upon interfacing with nanosensors (Figure 1f; Figure S7, Supporting Information). Fluorescence confocal micrographic analysis showed that nanosensor‐embedded leaves were not significantly different from leaves infiltrated with MES buffer; thus, the adverse effect of the nanosensors on leaves was negligible at the cellular level.

In addition, physiological properties were measured after nanoparticle infiltration into the leaves to determine whether the nanosensor‐embedded leaves could maintain the stomatal conductance, which is one of the crucial characteristics of the nanobionic VOC sensor plants. Leaf‐scale gas exchange was measured to show the changes in the stomatal conductance, the rate of net CO_2_ assimilation and the evapotranspiration at the leaf surface during the 3 h of observation. After nanosensor infiltration, the measured variables showed a course of changes over time but a similar temporal pattern to that of control group with buffer infiltration. The comparison of the net CO_2_ assimilation (photosynthesis – respiration, A_net_), evapotranspiration (E_T_), and stomatal conductance for water vapor (g_sw_) between the control and nanoparticle‐infiltrated groups did not reveal any significant differences (*p* > 0.05, one‐tailed *t*‐test) (Figure 1g).

### 2.2 Understanding VOC‐Mediated Interplant Communication Using Nanobionic Sensor Plants

**Figure 2:**
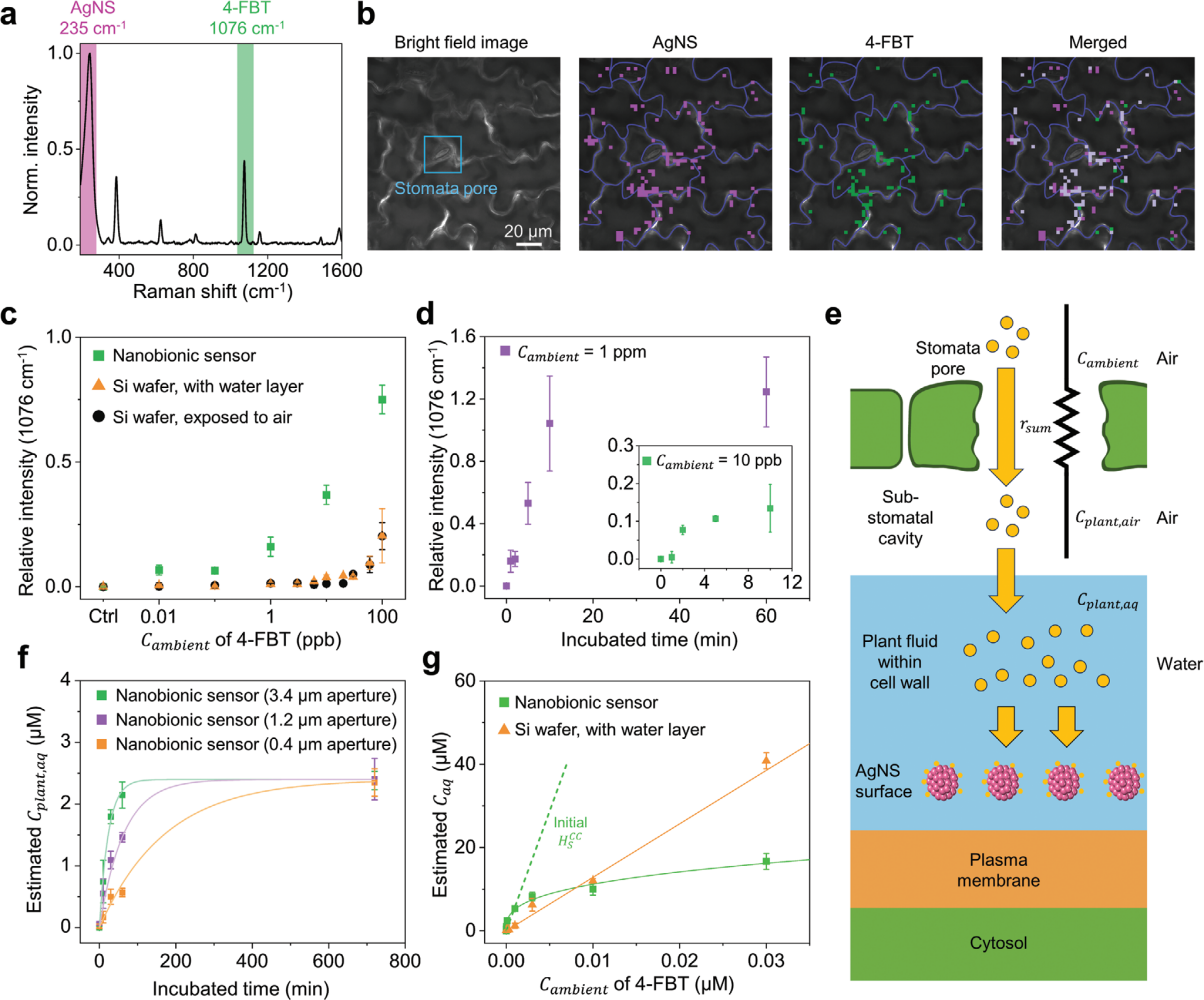
Demonstration of VOC sensing using a nanobionic sensor plant. a) Spectrum of the representative SERS signal from a nanobionic sensor plant after exposure to 10 ppb 4‐FBT. The SERS spectrum exhibits a band at 235 cm^−1^ specific to AgNS (purple) and a band at 1076 cm^−1^ specific to 4‐FBT (green). b) Bright‐field image of a representative plant leaf and its overlay images with Raman intensity maps for the AgNS signal and 4‐FBT signal from a 4‐FBT‐exposed plant leaf. The bright‐field image and Raman intensity maps were obtained near the stomata pore, which is highlighted with a blue box, using a 40× objective lens. Scale bar is 20 µm. Colocalized signals of AgNS and 4‐FBT were detected 3.5 times more frequently near the stoma (distance < 40 µm, 10.0% of pixels were colocalized, n: pixels with colocalization/whole pixels = 503/5024) compared to areas more than 40 µm away from the stomata (distance > 40 µm, 2.8% of pixels were colocalized, n: pixels with colocalization/whole pixels = 426/15072). Raman intensity maps were obtained from three individual clover plants (N = 3). c) Relative SERS intensities at 1076 cm^−1^ in various *C_ambient_* using nanobionic sensor plant (green) was compared with those from two kinds of silicon wafer‐based SERS substrates prepared with AgNS@PDDA, a substrate directly exposed to air (black) and a substrate with a water layer on it (orange). The SERS intensities were normalized to the intensity of the AgNS band at 235 cm^−1^ and are expressed as the relative intensity. d) Changes in Relative SERS intensities at 1076 cm^−1^ at the nanobionic sensor plant after various times of incubation with 4‐FBT at a *C_ambient_* of 1 ppm (purple). The inset graph displays changes in relative SERS intensity at 1076 cm^−1^ during incubation with 4‐FBT at a *C_ambient_* of 10 ppb (green). e) Illustration of the VOCs free diffusion occurred in the leaf of a nanobionic sensor plant. VOCs diffuse into the airspace inside plant leaves, which leads to resistance to *r_sum_*. The VOCs that pass through the stomata arrive at the cell wall covered with plant fluid and then bind to the SERS nanosensor surface. f) Effect of stomatal conductance on VOC diffusion rate. The stomatal aperture sizes were 1.2 µm (purple) and 0.4 µm (orange) after 5 and 50 µm ABA treatment, respectively, and 3.4 µm (green) without ABA treatment. The *C_ambient_* of 4‐FBT was 10 ppb for all measurements. The dots indicate the estimated *C*_*plant*,*aq*_ from calculation based on SERS intensity calibration curve, and solid curves correspond to the fitting according to Equation (5). g) Estimated concentration of aqueous 4‐FBT in a plant fluid of nanobionic sensor plant (green) and in water layer of a silicon wafer‐based SERS substrate (orange), in various *C_ambient_* of 4‐FBT. The orange line is a linear fitted line according to the wafer‐based SERS substrate data. The green curve is a logarithm‐fitted curve according to the measured data from the nanobionic sensor plant. The dashed green line is initial slope at nanobionic sensor plant with *C_ambient_* ranging from 1 × 10^−8^ to 1 × 10^−3^ µm (0.001–100 ppb), which corresponds to $H_{S}^{CC}$ for the nanobionic sensor plant, was 5750; this value is 4.5 times greater than that of wafer‐based SERS substrate (1280).

The SERS‐active nanosensor‐embedded white clover plant can immediately function as an optical nanosensor for detecting VOCs and is called a “nanobionic VOC sensor plant” in this study. To verify the localization of infiltrated nanosensors, SERS false‐color maps of nanosensor‐embedded clover leaves were obtained based on the intensity of the SERS band at 235 cm^−1^ (**Figure** **2**a,b); this corresponds to Ag···N stretching due to the interaction between the silver nanoshell surface and hexadecylamine, a reducing agent, added to form the silver nanoshell.^[^ ^42^ ^]^ Consistent with previous reports, SERS false‐color maps show that the nanosensors were found to be localized in the intercellular space of white clover leaves, specifically alongside the epidermal cell wall.^[^ ^31^ ^]^

To demonstrate its feasibility as a VOC sensor, a custom closed chamber was built using transparent acrylic plates. The nanobionic sensor plant was placed inside the chamber, and a droplet of 1 mm 4‐FBT ethanolic solution (12.5 µL) was placed 70 cm away from the plant in the same chamber; this setup provided gaseous 4‐FBT as a model VOC in the air, of which the ambient concentration of 4‐FBT in the chamber (*C_ambient_*) corresponded to be ≈10 ppb (parts per billion by weight). The 4‐FBT ethanolic solution completely evaporated after 1 min owing to its sufficiently high vapor pressure at room temperature (2.9 mmHg).^[^ ^43^ ^]^ In 2 min, the leaf of the nanobionic sensor plant exhibited the characteristic SERS signals of 4‐FBT (Figure 2a). The Raman intensity map based on the 1076 cm^−1^ band, which is the characteristic SERS band of 4‐FBT, indicated that vaporized 4‐FBT molecules passed through the stomata of the plant leaves and were immediately attracted by the SERS nanosensors located around the plant cell walls. The merged Raman intensity map shows that co‐localized signals of 4‐FBT SERS signals and SERS nanosensors themselves were detected near the stoma (distance < 40 µm) with a frequency of 10.0%, which is 3.5 times higher than in areas more than 40 µm away from the stomata (Figure 2b).

To evaluate the sensitivity of plants to VOCs, we placed the nanobionic sensor plant in the chamber for 1 h and exposed it to 4‐FBT in a range of *C_ambient_* from 10 ppt to 100 ppb. As *C_ambient_* increased, the SERS intensity at the leaves of the sensor plant gradually increased. Notably, the characteristic SERS bands of 4‐FBT were detectable on the leaves even when *C_ambient_* was decreased to the lowest level, 10 ppt (Figure 2c). This nanobionic VOC sensor plant detected a signal of 4‐FBT within a minute at 1 ppm of *C_ambient_* and within just 2 min at 10 ppb of *C_ambient_* (Figure 2d). This is a remarkable achievement that demonstrates its fast response time as well as high sensitivity.

To explain this exceptional sensitivity and investigate the role of the plant in VOC sensing ability, we observed the response of the SERS‐active nanosensors alone to VOCs excluding biological factors of the plant as a control experiment. The experiments were carried out using two types of SERS substrate and measured the SERS signals with varying *C_ambient_* of VOC. The first type of substrate was prepared by immobilizing the same AgNS@PDDA nanoparticles onto a silicon wafer and then exposing it to air (Figure S8, Supporting Information). The second type of substrate had a water layer added on top of the first type, mimicking the interaction between liquid layer and nanosensors at the SERS nanosensor‐plant interface. We then placed each type of substrate in a separate closed chamber containing varying concentrations of 4‐FBT for 1 h to obtain the measurements. When the substrate was directly exposed to air without water layer, the SERS signal was detected at *C_ambient_* above 30 ppb. When the substrate, covered with a water layer, was exposed to air, the SERS detection sensitivity could be lowered to *C_ambient_* of 10 ppb (Figure 2c). These results indicated that the water layer on the SERS nanosensor particles played a vital role in the interaction between the VOC molecules and the SERS substrate. The water layer facilitated more favored adsorption of VOCs than adsorption onto a dry SERS substrate, which indicated that VOCs underwent a decrease in thermodynamic free energy when they approached a solid surface covered with a water layer.^[^ ^44^ ^]^ Subsequently, this enabled the VOCs to spontaneously dissolve into the water layer,^[^ ^45^ ^]^ concentrating the VOCs at the proximity of SERS hot spots. An air–water interface within the leaf of the nanobionic VOC sensor plant contributes to the high sensitivity of detecting low concentrations of VOCs. However, further research is required to fully explain its sensitivity in detecting 4‐FBT at 10 ppt of *C_ambient_*, which is 1000 times lower than that of the SERS substrate with a water layer (Figure 2c). These findings have significant implications, as the high sensitivity of nanobionic sensor plants cannot be attributed solely to the sensitivity of nanosensors. This suggests that VOC condensation takes place within the plants, a critical factor in explaining the exceptional sensitivity.

To gain a deeper understanding of the VOC detection in plants; we have focused on the biological condensation of VOCs within the plants and constructed a mathematical model to estimate the concentration of VOCs in the plant fluid. The nanobionic VOC sensor plant operates based on the process of VOC condensation after free diffusion, which can be described using a mathematical model using the following assumptions: 1) VOCs enter plant leaves through free diffusion, 2) the diffused VOCs are dissolved in plant fluid through chemical equilibrium (Figure 2e).

To calculate the flux of the VOCs entering the stomata, the resistance that gas molecules experience when they pass through stomata need to be considered. The amount of VOCs in the air was assumed to be sufficient; thus, *C_ambient_* of VOCs remained constant throughout the diffusion process. This resistance is known as the “stomatal resistance (*r_s_*)” or its reciprocal form, the stomatal conductance (*g_s_*).^[^ ^46^ ^]^ The conductance value can be approximated using Fick's first law^[^ ^47^ ^]^ as follows:

(1) $$\begin{array}{ccc} J & = & -D\cdot \frac{dC}{dx}\approx -D\frac{\Delta C}{l}=g\left(C_{ambient}-C_{plant,air}\right)\\ & = & \frac{\left(C_{ambient}-C_{plant,air}\right)}{r} \end{array}$$

where *J* is the flux, *D* is the diffusion coefficient, *C* is the concentration, $\frac{dC}{dx}$ is the concentration gradient of VOC, and *l* is the effective diffusion length through the stomata. *C*_*plant*,*air*_ is the concentration of gaseous VOC within the airspace inside the plant leaf. The stomatal conductance, *g_s_*, can be calculated by the following equation:^[^ ^47^ ^]^

(2) $$g_{s}=\frac{DmA_{s}}{\left(L+2\times end\;correction\right)}$$

where *m* is the stomatal density, *A_s_* is the area of the stomatal pore, and *L* is the length of the stomatal pore. *A_s_* was determined assuming that the stomatal pore has the shape of an ellipse, where the major axis remained constant and the minor axis varied according to the opening of the stomatal pore (Figure S9, Supporting Information). Additionally, an equation was established to estimate *C*_*plant*,*air*_ over time until the diffusion process of VOC in the plant reaches equilibrium. Instead of stomatal conductance (*g_s_*), total conductance (*g_sum_*) was applied to consider the total resistance in the pathway of VOC movement. Detailed information is available in the “Calculation of the VOC concentration inside stomata” section of the Supporting Information.

(3) $$C_{plant,air}\left(t\right)\approx C_{ambient}\cdot \left(1-exp\left(\frac{A_{c}g_{sum}t}{V_{plant,air}}\right)\right)$$

where *A_c_* is the area of the virtual aerial column through which the VOC passes after the stomatal pore, *t* is time duration of VOC exposure, and *V*_*plant*, *air*_ is the volume of the airspace inside the plant leaf (Figure S10, Supporting Information). After diffusion, the VOC molecules that passed through the stoma arrive at the cell wall with the plant fluid (Figure 2e). The SERS nanosensor are located along the cell walls,^[^ ^31^ ^]^ and possibly covered with the plant fluid. These processes can be expressed as follows:

(4) $$A\left(plant,air\right)\Leftrightarrow A\left(plant,aq\right)\;K_{water/air}=H_{S}^{CC}=\frac{C_{plant,aq}}{C_{plant,air}}$$

where *A* stands for VOC specimens present in each specific state, *K*_*water*/*air*_ is a partition function of the VOC molecule between the gas and aqueous phases; specifically, it is a dimensionless Henry's constant, $H_{S}^{CC}$. The gas phase of VOCs becomes dissolved in plant fluid, and then approaches the SERS sensors, thus generating the SERS signals. Detailed information is available in the “VOC binding at SERS nanosensor” section of the Supplementary Information.

Based on our previous consideration of the free diffusion process and equilibrium, concentration of VOCs dissolved in the plant fluid layer (*C*_*plant*,*aq*_) can be estimated using the following equation:

(5) $$\begin{array}{ccc} C_{plant,aq,\;t} & = & C_{plant,air,\;t}\cdot H_{S}^{CC}\approx C_{ambient}\cdot \left(1-exp\left(\frac{A_{c}g_{sum}t}{V_{plant,\;air}}\right)\right)\\ & & \cdot H_{S}^{CC} \end{array}$$

To verify this model experimentally, we obtained the concentration profile of the VOCs in the plants, *C*_*plant*,*aq*_, by SERS measurement (Figure S11, Supporting Information). When the nanobionic VOC sensor plant was placed in a closed chamber where the *C_ambient_* of 4‐FBT is 10 ppb, estimated *C*_*plant*,*aq*_ sharply increased as the VOC diffused into the leaf, then the diffusion rate gradually decreased until *C*_*plant*,*aq*_ eventually reached the *C_ambient_* (Figure 2f). The model indicates that *C*_*plant*,*aq*_ is directly affected by stomatal conductance (Equations (3) and (5)); therefore, abscisic acid (ABA), which is a plant hormone that induces stomatal closure,^[^ ^48^ ^]^ was administered to the leaves to adjust the stomatal conductance corresponding to the diffusion rate (Figure 2f). A decrease in the stomatal aperture leads to lower stomatal conductance, which in turn reduces the rate of VOC uptake by plants (Table S1, Supporting Information). The initial stomatal aperture of the white clover plants was 3.4 µm, and for the plants treated with 5 and 50 µm ABA, the stomatal aperture decreased to 1.2 and 0.4 µm, respectively. Since this stomatal aperture size remained constant for 24 h following ABA treatment, the stomatal conductance was adequately controlled during 12 h of SERS signal measurement (Figure S5, Supporting Information). In the experiments, the VOC uptake rate of control plants was compared with those of plants with reduced stomatal aperture sizes. Compared to the control plants, plants with a 1.2 µm stomatal aperture had a 68% uptake rate, and plants with a 0.4 µm stomatal aperture had a 26% uptake rate at the 1‐h time point (Figure 2f). The results from the experiment showed a close correlation with the diffusion model plot we described (adj. R^2^ = 0.9876). This model explains how VOC accumulates in the plant fluid over time and *C*_*plant*,*aq*_ reaches a certain value, $C_{\mathrm{ambi}\mathrm{ent}}\cdot H_{S}^{\mathrm{CC}}$, regardless of the stomata conductance. The close agreement between experimental results and the diffusion model plot provides strong evidence supporting the feasibility of our established model.

The sensitivity can be affected by VOC solubility, thus, we derived $H_{S}^{CC}$ from the diffusion model on Equation (5) to compare the solubility of VOCs in the nanobionic sensor plants and SERS substrates with a water layer (Figure 2g). The $H_{S}^{CC}$ for the SERS substrate with a water layer was 1280, while the $H_{S}^{CC}$ for the nanobionic sensor plant was 5750, which is 4.5 times greater than that of the non‐plant SERS substrate covered with the water layer. The difference in $H_{S}^{CC}$ suggests that plant fluid can dissolve VOCs more effectively than water. Therefore, nanobionic sensor plants respond more sensitively to ambient VOCs. As we assumed, VOC condensation occurs within nanobionic sensor plant leaves using a living plant system, enhancing the nanobionic sensor plant's ability to detect airborne VOCs at extremely low *C_ambient_*. The following factors could explain the higher $H_{S}^{CC}$ and VOC condensation in plants. First, VOCs may be more soluble in plant fluids than in pure water since plant fluid contains hydrophobic organic compounds such as lipids, wax, and lignin,^[^ ^49^ ^]^ enhancing the solvation of VOCs.^[^ ^50^ ^]^ Furthermore, the transpiration process cools the substomatal cavity through water evaporation,^[^ ^51^ ^]^ lowering the local temperature by up to 3 °C^[^ ^52^ ^]^ and possibly increasing gas solubility.

Interestingly, this effect was particularly only observed at low concentrations of VOCs with low *C_ambient_* under 0.001 µm (100 ppb). The response of the nanobionic sensor plant was not linearly proportional to the *C_ambient_* (Figure 2g), estimated *C*_*plant*, *aq*_ was even lower than that of water above 0.01 µm (1000 ppb) of *C_ambient_*. Since the VOC concentration needed for plant intercommunication is very low, plants can become overwhelmed if too many compounds are present in the air. In response, plants have a defense system that can be triggered by high *C_ambient_* of airborne molecules.^[^ ^53^ ^]^ This system includes generating reactive oxygen species (ROS), closing stomata, increasing diffusive resistance, and decreasing the photosynthesis rate.^[^ ^54^ ^]^ When the *C_ambient_* of VOC exceeds several hundred ppb, these responses are activated, and the VOC uptake by plants is reduced.^[^ ^55^ ^]^ A decrease in the transpiration rate can also lower the solubility of the VOCs in the plant fluids by reducing the local cooling effects. As a result, estimated *C*_*plant*, *aq*_ is relatively lower than that in water. The natural defense system of plants can protect themselves from some gaseous hazards by regulating the absorption of VOCs through physiological reactions when exposed to excessively high *C_ambient_* of VOCs that could harm their structure. Therefore, the plants can remain healthy and effectively monitor the ambient VOC without withering.

**Figure 3:**
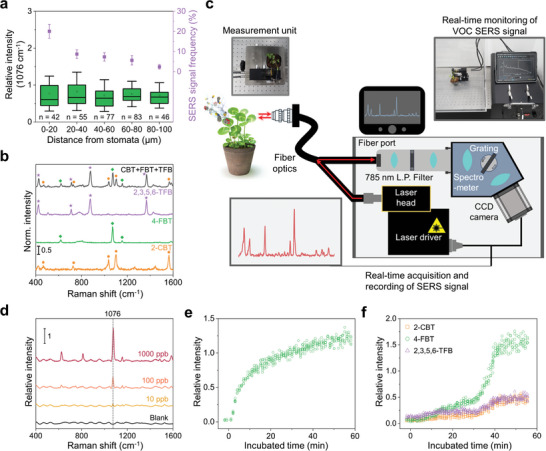
Applicability of the nanobionic sensor plant as a VOC sensor. a) SERS intensity and signal detection frequency at the nanobionic sensor plant depending on the distance from the stomata. The SERS intensities are presented as a box plot where the box ranges from the first to third interquartile; here, the horizontal line and small rectangle indicate the median and mean, respectively, and the whiskers extend to ±1× standard deviation. The SERS signal detection frequencies are depicted using purple dots; these indicate the percentage of pixels where the SERS signals were detected from all pixels in the SERS intensity map. Measurements were conducted on the nanobionic sensor plant after incubation with 100 ppb 4‐FBT. b) Multiplex detection of three types of VOCs. SERS spectra were obtained for each VOC, such as 0.1 ppm 2‐CBT (orange), 0.1 ppm 4‐FBT (green), 0.1 ppm 2,3,5,6‐TFB (purple) and their gaseous mixture, 2‐CBT+4‐FBT+2,3,5,6‐TFB (gray). The concentration of each molecule was 0.5 ppm for 2‐CBT, 0.5 ppm for 4‐FBT, and 0.2 ppm for 2,3,5,6‐TFB in the mixture. The symbols indicate the characteristic bands contributed by 2‐CBT (orange circle), 4‐FBT (green square), and 2,3,5,6‐TFB (purple star) c) Illustration of the experimental setup for real‐time monitoring of VOCs using a nanobionic sensor plant with a customized portable Raman device. d) SERS spectra of 4‐FBT at various airborne concentrations measured on a nanobionic sensor plant with a portable Raman device. The SERS acquisition time was 60 s for the control and nanobionic sensor plants with 10 ppb 4‐FBT exposure and 1 s for the nanobionic sensor plants with 100 and 1000 ppb 4‐FBT exposure. e) Continuous monitoring of SERS signal intensity at 1076 cm^−1^ on a nanobionic sensor plant, recorded during incubation with 4‐FBT with *C_ambient_* of 1 ppm. 4‐FBT exposure was started from 0 min, and 5 spectra were taken every minute with 1 s acquisition. f) Continuous monitoring of SERS signal intensity on a nanobionic sensor plant recorded during incubation with a gaseous mixture of 2‐CBT, 4‐FBT, and 2,3,5,6‐TFB. The concentration of each molecule was 5 ppm for 2‐CBT, 5 ppm for 4‐FBT, and 2 ppm for 2,3,5,6‐TFB in the mixture. VOC exposure began at 0 min, and 5 spectra were taken every minute with a 1 s acquisition time. The SERS intensities of the characteristic bands of each VOC were monitored: 1103 cm^−1^ for 2‐CBT (orange), 1076 cm^−1^ for 4‐FBT (green), and 880 cm^−1^ for 2,3,5,6‐TFB (purple). The SERS intensities were normalized to the intensity of the AgNS band at 235 cm^−1^ and are expressed as the relative intensity. The laser power was 7.5 mW for all measurements.

To understand VOC distribution in the leaf after VOC uptake, wide‐area SERS intensity maps were obtained for separated areas in a single leaf of a nanobionic sensor plant (Figure S12, Supporting Information). The SERS intensities from each area showed a similar trend, exhibiting no significant difference in their mean intensities. Then, to investigate whether the SERS signal intensities were affected by the distance from the stomata pore, we obtained the SERS signals and classified them based on their distance from the stomata pore (**Figure** **3**a). The SERS signals were more frequently detected near the stomata pore; however, their intensities showed no significant differences according to distances from the stomata. Based on these experiments, the nanobionic sensor plant produced a consistent level of SERS signal within a leaf, regardless of the measurement location. In a chamber, a nanobionic sensor plant is placed 70 cm away from the analyte, which allows VOCs to disperse uniformly in the air before reaching the nanosensor particles. Even if VOCs enter through the stomata away from the nanoparticle‐infiltrated region within the same leaf, VOCs can be diffused through the air space inside the leaf and eventually reach the nanosensor particles. The time it takes for gas molecules to diffuse over a specific distance can be calculated using the equation derived from Fick's law:

(6) $$t=\frac{L^{2}}{2D}$$

where L is diffusion length, which reaches a maximum of 7 mm in the clover leaf. Diffusion coefficient of 4‐FBT in free air (*D*_4 − *FBT*,  *air*_) was calculated to be 3.66 × 10^−6^ m^2^ s^−1^ (S1, Supporting Information), while diffusion coefficient within the air space of the leaf is estimated to be 10–25% of the value in free air.^[^ ^56^, ^57^ ^]^ Assuming that the diffusion coefficient for 4‐FBT in the air space inside the clover leaf is ~3.66 × 10^−7^–9.15 × 10^−7^ m^2^ s^−1^ (10–25% of the free air value), it will take ~27–67 s for the gaseous 4‐FBT to disperse evenly throughout the entire clover leaf. Consequently, within 1 min, the absorbed VOCs in the leaf are expected to distribute and this results in a consistent SERS signal throughout the leaf, independent of the measurement location. To demonstrate the multiple VOC detection in plants, mimicking a real circumstance of interplant communication with multiple signaling molecules, we placed the nanobionic sensor plant with three different volatile compounds, 2‐chlorobenzenethiol (2‐CBT), 4‐FBT and 2,3,5,6‐tetrafluorobenzenethiol (2,3,5,6‐TFB) in a closed chamber. When the ethanolic solutions of these three benzene thiols were placed with the nanobionic VOC sensor plant, the characteristic SERS bands of each compound were collected from the nanosensor‐embedded leaves. The SERS signals showed distinct bands for each benzene thiols; for instance, 2‐CBT had peaks at 729, 1038, 1103, and 1567 cm^−1^; 4‐FBT had peaks at 621, 1076, and 1156 cm^−1^, and 2,3,5,6‐TFB had peaks at 713, 880, 1368, and 1627 cm^−1^. These three chemicals were individually placed as ethanolic solutions next to the nanobionic sensor plant to provide a mixture of VOCs in the environment inside the chamber. With multiple VOCs in the air, the nanobionic sensor plant instantaneously monitored multiple SERS spectra corresponding to 2‐CBT, 4‐FBT, and 2,3,5,6‐TFB (Figure 3b).

Since engineering plants using nanobiotechnology is a technique that is independent of species, we conducted identical experiments on watercress plants to see if a similarly sized plant could serve as a VOC sensor with comparable sensitivity. The results from the nanosensor‐embedded watercress plants were equivalent to those from the nanobionic clover plants (Figure S13, Supporting Information), 4‐FBT *C_ambient_* of 10 ppt being detectable. The similarity in their VOC sensing abilities can be attributed to their comparable stomatal aperture size and density (Table S2, Supporting Information). These findings suggest that adapting the plant nanobionic approach allows for highly sensitive VOC detection, regardless of the plant species used in nanobionic engineering.

### 2.3 VOC Detection with a Portable Raman Spectrometer

Given that plants respond to external stimuli often within a few minutes,^[^ ^58^, ^59^ ^]^ with their reactions frequently changing from moment to moment,^[^ ^60^ ^]^ a real‐time sensor capable of instantly detecting these responses is essential for effectively observing plant's behavior and elucidate the biological processes involved in signaling molecule generation. Moreover, the sensor's capability for continuous monitoring enhances its practical application in detecting trace airborne molecules in on‐field situations.^[^ ^61^, ^62^ ^]^ Thus, we constructed a customized portable Raman spectrometer to demonstrate the feasibility of nanobionic sensor plants for on‐field VOC sensing (Figure 3c). This portable Raman device is equipped with optical fiber‐based optics and a NIR (785 nm) laser source and contains a Raman spectrometer with a spectral resolution of 2 cm^−1^. The device has an optical probe and an objective lens (×10). Using an objective lens and single‐mode NIR laser helps to minimize the background plant autofluorescence and improves the SERS measurement sensitivity. This portable Raman device does not have a vision component to make it smaller and more suitable to field study. As mentioned earlier, the position at which a measurement is taken on a nanobionic sensor plant has a negligible impact on the SERS signal intensity (Figure 3a; Figure S12, Supporting Information). Therefore, if SERS signals are collected from a single spot on a leaf, they can be equivalent to those obtained from the entire leaf, which can overcome the difficulty of finding the point where the SERS signal is the strongest without a vision component. The portable Raman device has a cylindrical Raman collection volume with an approximate diameter of 40 µm and height of 105 µm, as estimated from the measured beam size. Therefore, by utilizing the consistent SERS signals generated by the nanobionic sensor plant, a portable Raman device could acquire integrated SERS signals with fine spectral quality from a wide leaf area in a single measurement. By leveraging the advantages of the nanobionic sensor plant, a portable Raman device could detect the SERS signals from the nanobionic sensor plant with a high signal‐to‐noise ratio of 55 for 4‐FBT *C_ambient_* of 1 ppm without requiring a vision component (Figure 3d). The quality of the signal is comparable to the SERS signal obtained from Raman microscopy, which showed a signal‐to‐noise ratio of 148 for the same concentration of airborne 4‐FBT. Airborne 4‐FBT SERS signals were detected from the nanobionic sensor plant down to *C_ambient_* of 10 ppb, indicating a sensitivity comparable to that of Raman microscopy. This device facilitated continuous monitoring of the SERS signals from the nanobionic sensor plant. When the sensor plant, 4‐FBT droplet, and measurement unit of the portable Raman device are placed together within a closed chamber, the SERS spectrum of the sample is recorded every minute for 1 h, with 1 s of SERS acquisition time and 7.5 mW of laser power (Figure 3e). Combined with the portable Raman device, the nanobionic sensor plant could instantly detect VOCs by observing the changes in the SERS signal; this shows the potential application of the nanobionic sensor plant as an on‐field VOC sensor capable of continuously monitoring VOCs.

The portable Raman spectrometer has a high resolution that allows it to detect each characteristic band from multiple VOCs in a nanobionic sensor plant. When the nanobionic sensor plant was placed in a closed chamber containing a mixture of two or three VOCs, the sensor was able to detect all the unique bands of the VOCs, allowing for their identification (Figure S14, Supporting Information). This ability to detect multiple VOCs is comparable to the capabilities of a Raman microscope. Additionally, by combining a nanobionic sensor plant with a portable Raman device, continuous monitoring of VOCs can be achieved. The SERS spectrum from the nanobionic sensor plant was recorded every minute, and the intensities of the characteristic bands of each VOC, such as 1103 cm^−1^ for 2‐CBT, 1076 cm^−1^ for 4‐FBT, and 880 cm^−1^ for 2,3,5,6‐TFB, were monitored in real‐time (Figure 3f; Figure S15, Supporting Information). After ~5 min of incubation, a discernible signal for 4‐FBT appeared, followed by signals for 2‐CBT and 2,3,5,6‐TFB after 10 min of incubation. The signal intensities gradually increased until the 40‐min mark, when the intensities of those signals reached their maximum. When the nanobionic sensor plant was placed with a mixture of VOCs, the SERS signals appeared at a slower rate than when the plant was incubated with a single type of VOC. For a single VOC, the SERS signal appeared in only 3 min (Figure 3e). The response times could be attributed to the competitive interaction of the VOCs with the nanosensor surface since these VOCs share binding sites on the surface. These results show that nanobionic sensor plants are capable of simultaneously monitoring multiple VOCs, precisely identifying their composition, and tracking their occurrence by analyzing specific SERS signals of each VOC.

### 2.4 Monitoring of the VOC‐Mediated Plant Intercommunication

**Figure 4:**
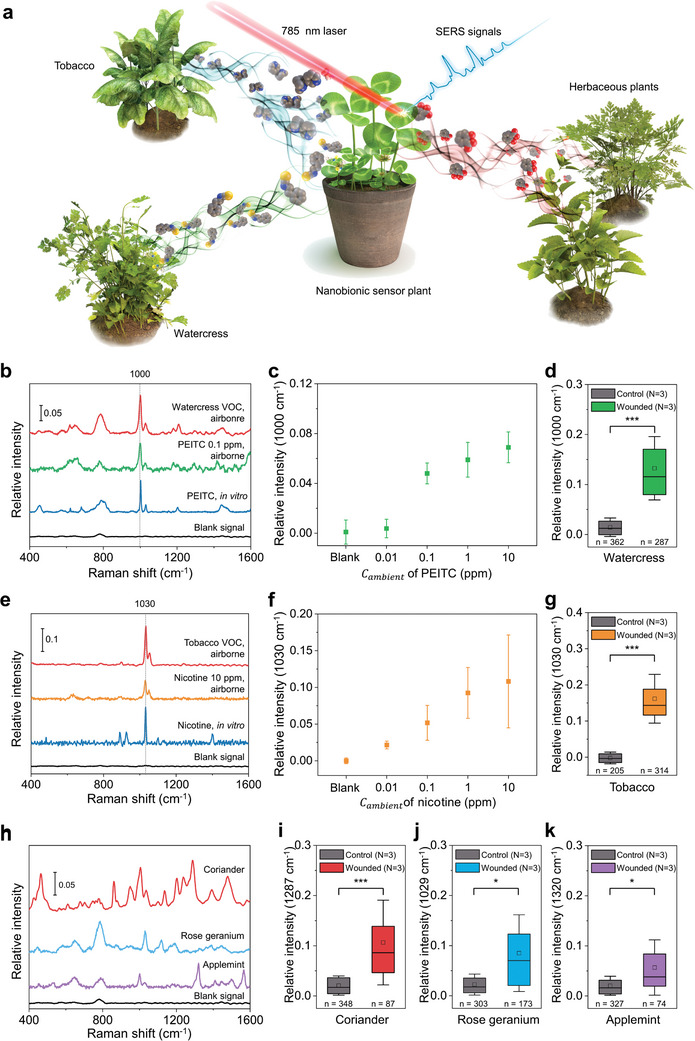
Plant signaling VOC detection with a nanobionic sensor plant. a) Illustration of monitoring VOC‐mediated interplant communication by using a nanobionic sensor plant. b) Representative SERS spectra detected in the sensor plants after placing with the wounded watercress plants (red), in the sensor plants after incubating with phenethyl isothiocyanate (PEITC) *C_ambient_* 0.1 ppm (green), in the solution of 1 mm PEITC (blue), and the sensor plant (AgNS@PDDA‐infiltrated clover) without any analyte (black). c) PEITC ambient concentration dependence of the SERS intensity at 1000 cm^−1^ measured from a nanobionic sensor plant. Data are presented as the mean±s.d. based on three individual experiments at each time point (*N* = 3). d) Comparison of the SERS intensity of PEITC between the wounded watercress (green) and control watercress plants without wounds (gray). The intensities of the band at 1000 cm^−1^, corresponding to PEITC, were measured in three individual experiments for each group. The SERS signal intensities are presented in a box plot; here, the box ranges from the first to third interquartile, with the horizontal line and small rectangle indicating the median and mean, respectively, and the whisker indicating ±1× the standard deviation. The number of spectra in each group is presented as n. The asterisks indicate values that are significantly different from the control (^***^ *p* < 0.0001, two‐sided Mann–Whitney U test). e) Representative SERS spectra detected in the sensor plant after placing with the wounded tobacco plants (red), in the sensor plant after incubating with nicotine *C_ambient_* 10 ppm (orange), in the solution of 1 mm nicotine (blue), and the sensor plant (AgNS@PVA‐infiltrated clover) without any analyte (black). f) Nicotine ambient concentration dependence of the SERS intensity at 1030 cm^−1^ measured from a nanobionic sensor plant. The data are represented as mean±s.d. based on three individual experiments at each time point (N = 3). g) Comparison of the SERS intensity of nicotine between the wounded tobacco (orange) and control tobacco plants without wounds (gray). The intensities of the nicotine band at 1030 cm^−1^ were measured in three individual experiments for each group. The SERS signal intensities are presented in a box plot where the box ranges from the first to third interquartile; here, with the horizontal line and small rectangle indicate the median and mean, respectively, and the whisker indicates ±1× the standard deviation. The number of spectra in each group is presented as n. The asterisks indicate values that are significantly different from the control (^***^ *p* < 0.0001, two‐sided Mann–Whitney U test). h) Representative VOCs SERS spectra detected in the sensor plants after placing with three kinds of herbs. The VOC signals from the wounded coriander (*Coriandrum sativum*, red), wounded rose geranium (*Pelargonium graveolens*, blue), and wounded applemint (*Mentha Suaveolens*, purple) were monitored. i–k) Comparison of SERS intensity of VOCs between the wounded herbs and control herbs without wounds (gray). Each plot represents the SERS signal from different plants, namely, coriander (red, i), rose geranium (blue, j), and applemint (purple, k). The SERS intensities were measured in three individual experiments for each group. SERS signal intensities are presented in a box plot where the box ranges from the first to third interquartile; here, the horizontal line and small rectangle indicate the median and mean, respectively, and the whisker indicates ±1× standard deviation. The number of spectra in each group is represented as n. The asterisks indicate values that are significantly different from the control (^*^ *p* < 0.05, ^**^ *p* < 0.001, ^***^ *p* < 0.0001, two‐sided Mann–Whitney U test). All the SERS intensities were normalized to the intensity of the AgNS band at 235 cm^−1^ and are expressed as the relative intensity.

Plants produce signaling molecules to maintain homeostasis, defend themselves from risk factors, and inform nearby plants on external stimuli.^[^ ^63^ ^]^ Plants are known to generate a variety of signaling molecules, which are highly variable among plant specimens.^[^ ^64^ ^]^ Typical signaling molecules for interplant communications are delivered through the air and are emitted and transmitted to surrounding plants through the stomata pores and cuticles.^[^ ^65^ ^]^ However, technology for individually detecting and identifying volatile molecules in airborne states has not yet been developed.^[^ ^66^ ^]^ As mentioned earlier in this manuscript, we showed that a nanobionic sensor plant could work as an autonomous VOC sensor capable of detection and identification. This strategy can be applied to detect and analyze VOC‐mediated interplant communications (**Figure** **4**a). We chose to focus on the analytes, phenethyl isothiocyanate (PEITC)^[^ ^67^, ^68^, ^69^, ^70^ ^]^ and nicotine,^[^ ^71^, ^72^, ^73^ ^]^ that have been previously identified using GC‐MS, a standard method for analyzing VOCs, to demonstrate reliability of our nanobionic plant sensors. PEITC is abundant in cruciferous vegetables including watercress^[^ ^67^ ^]^ and is known to be produced as a defense mechanism against external physical stimuli, which such as predation and pest attack.^[^ ^69^ ^]^ To verify that the detected VOC was PEITC, the nanobionic sensor plant was placed with PEITC *C_ambient_* of 0.1 ppm for 60 min, and the characteristic SERS bands of PEITC at 1000, 1030, and 1210 cm^−1^ in the sensor plant were identical to the SERS spectrum collected from the PEITC solution (Figure 4b). The relative SERS intensity of PEITC at 1000 cm^−1^ increased with PETIC concentration in the range of *C_ambient_* 0.01–10 ppm (Figure 4c). When a wounded watercress plant, which had scratches on its leaves, was placed next to a nanobionic sensor plant, the SERS signals of PEITC emitted from the wounded watercress were detected by the sensor plant (Figure 4b,d). Before monitoring the VOC‐mediated wound signals between the plants, we ensured that no PEITC molecules were present in the clover leaves, even those that were physically wounded (Figure S16, Supporting Information). The SERS signals from PEITC were not observed in the clover leaves, as these molecules were not produced within the sensor plants. Instead, the PEITC signals were detected from the nanosensor‐embedded clover plants, where the gaseous PEITC molecules were transmitted through the air from the wounded watercress plants to the sensor plants.

Nicotine is a signaling molecule found in tobacco plants and is released into the atmosphere in response to external stimuli, such as physical damage.^[^ ^71^, ^72^, ^73^, ^74^ ^]^ To monitor nicotine, AgNS@PVA was introduced into clover leaves to detect airborne nicotine and was expected to interact with nicotine via hydrogen bonding with the hydroxyl group of the PVA polymer.^[^ ^38^, ^39^ ^]^ Compared with other nanosensors such as AgNS@PDDA or AgNS@PAA, AgNS@PVA produced the best SERS spectrum for the nicotine molecules (Figure S17a, Supporting Information). The clover plant integrated with AgNS@PVA was placed next to the ethanolic nicotine solution, leading to nicotine *C_ambient_* of 10 ppm. The SERS bands of airborne nicotine detected from the nanobionic sensor plant appeared at 1030 and 1050 cm^−1^; these bands were identical to those from the leaves infiltrated with aqueous nicotine solution (Figure 4e). The SERS spectrum collected from the inside the plant was slightly different from the SERS spectrum of aqueous nicotine; the difference included the absence of the bands at 924 cm^−1^ and an increase in the intensity at 1050 cm^−1^, possibly due to the much more complex chemical composition in the plant environment. With varying nicotine *C_ambient_*, the SERS signal intensity collected from the AgNS@PVA‐embedded plant increased with increasing in nicotine *C_ambient_* (Figure 4f). When the nanobionic sensor plant was present next to the wounded tobacco plants, a nicotine SERS signal was detected from the sensor plant (Figure 4e,g). Such signals were not detected in the nanobionic sensor plant after incubating with control tobacco plants with no wounds. These results indicated that nicotine was released from tobacco plants as a signaling molecule in response to the physical wounds and that the surrounding plants were able to collect these signals.

VOCs from different plant species were further detected to demonstrate the versatility of nanobionic sensor plants for monitoring different signaling molecules among the different plants (Figure 4h). Herbaceous plants were selected for VOC detection because they are known to emit a wide range of volatiles in response to external stimuli. Some of those VOCs are aromatic derivatives,^[^ ^75^ ^]^ which are considered to have large Raman cross‐sections and are thus effectively detected with the SERS measurements. The SERS spectra from the nanosensor‐embedded clover plant were collected after it was placed next to several wounded herbs, such as coriander (*Coriandrum sativum*), rose geranium (*Pelargonium graveolens*), and applemint (*Mentha Suaveolens*). Each herb plant emitted VOCs with unique SERS spectra that were distinguishable from each other. Representative peaks appeared at the following wavenumbers: 465, 1004, 1287, and 1479 cm^−1^ for the VOCs from coriander; 1030, 1119, and 1626 cm^−1^ for the VOCs from rose geranium; and 645, 1000, 1320, and 1566 cm^−1^ for the VOCs from applemint. The bands at 860 cm^−1^ (ring breathing, for coriander), 1000 ∼ 1030 cm^−1^ (ring breathing, 1004 cm^−1^ for coriander, 1030 cm^−1^ for rose geranium, and 1000 cm^−1^ for applemint) and 1180–1210 cm^−1^ (ring vibration, 1203 cm^−1^ for coriander and 1189 cm^−1^ for rose geranium) were attributed to benzene rings of aromatic compounds.^[^ ^76^, ^77^, ^78^ ^]^ The relative intensity of the SERS signals from the VOCs emitted from wounded herb plants was significantly greater than that from unwounded plants (Figure 4i–k); thus, the SERS signals collected using the nanobionic sensor plant represent the chemical signals between the plants. Although the exact VOC compositions have not been specified, the SERS spectra collected from herb plants using the nanobionic VOC sensor plant showed the presence and visualization of VOC‐mediated plant intercommunications.

### 2.5 Detection of Various Airborne Chemicals

**Figure 5:**
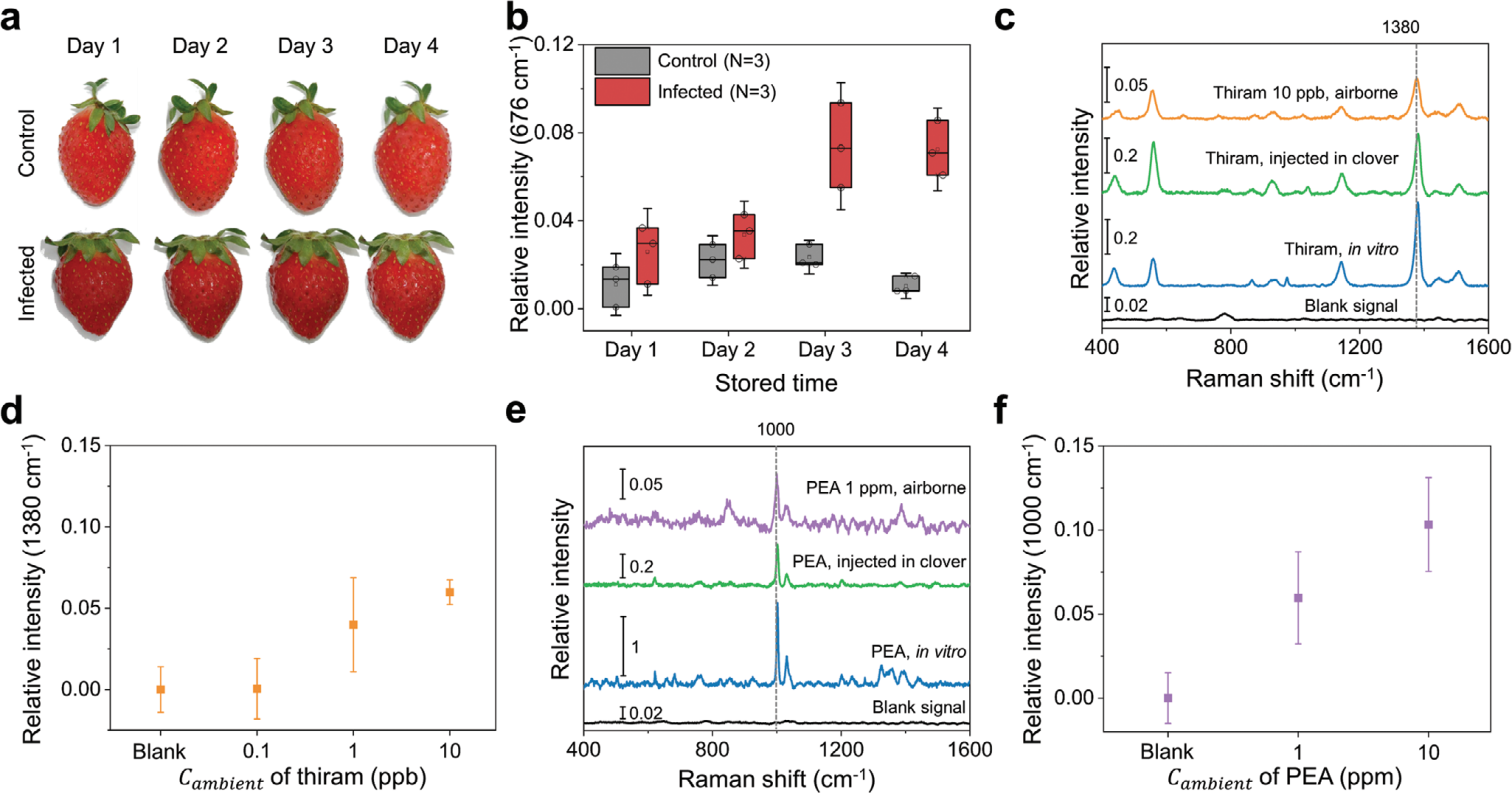
Various volatile chemical detection with nanobionic sensor plants. a) Images of strawberry fruits from the control group (upper row) and the group infected with gray mold (lower row), captured 1 to 4 days after inoculation. Both groups were sterilized, and the infected groups were treated with 10^6^ PFU of gray mold (*Botrytis cinerea*). Fruits from both groups were kept at 4 °C to minimize other factors affecting decomposition other than fungal infection, and no significant changes in appearance were observed upon visual observation until 4 days after inoculation b) Comparison of relative SERS signal intensity of methanethiol between the volatiles from the strawberry fruits in the control group (gray) and those from the gray mold‐infected strawberry fruits (red). SERS signals were collected from three individual experiments for each group. The data are presented as a box plot where the box ranges from the first to third interquartile; here, the horizontal line and small rectangle indicate the median and mean, respectively, and the whisker indicates ±1.5× the standard deviation. c) Representative SERS spectra detected in the sensor plant after incubating with thiram *C_ambient_* 10 ppb (orange), in the sensor plant after thiram infiltrated into clover leaves (green), in the solution of 10 µm thiram (blue), and in the sensor plant (AgNS@PDDA‐infiltrated clover) without any analyte (black). d) Thiram ambient concentration dependence of the relative SERS intensity at 1380 cm^−1^ measured from a nanobionic sensor plant. The data are presented as mean±s.d. based on three individual experiments at each point (*N* = 3). e) Representative SERS spectra detected in the sensor plant after incubating with phenethylamine (PEA) *C_ambient_* 1 ppm (purple), in the sensor plant after PEA injected into clover leaves (green), in the solution of 100 mm PEA (blue), and in the sensor plant (AgNS@PAA‐infiltrated clover) without any analyte (black). f) PEA airborne concentration dependence of the SERS intensity at 1000 cm^−1^ measured from a nanobionic sensor plant. The data are presented as mean±s.d. based on three individual experiments at each time point (*N* = 3). All the SERS intensities were normalized to the intensity of the AgNS band at 235 cm^−1^ and are expressed as the relative intensity.

The detection and analysis of VOCs using a nanobionic sensor plant provides unique advantages because the sampling process is rapid and non‐invasive, requires no additional devices, and can be carried out in real‐time. We investigated the ability of the nanobionic sensor plant to detect VOCs for human benefit in a variety of fields. First, VOC detection for monitoring food spoilage was demonstrated. We monitored the SERS signals of VOCs emitted from strawberry fruits infected by *Botrytis cinerea*., also known as gray mold; this is a common pathogen that affects plants, including strawberries, without causing any symptoms until the fruit ripens.^[^ ^79^ ^]^ Gray mold continuously grows even after harvest and affects fruit ripening and postharvest decay; thus, it is a major concern affecting the fresh product quality. To minimize other factors affecting decomposition in addition to fungal infection, *B. cinerea* was applied to the surface of sterilized strawberries, and the infected fruits were kept at 4 °C (**Figure** **5**a). When the nanobionic sensor plant was placed with the infected strawberry fruits in the chamber, the nanosensor‐embedded clover leaves showed SERS bands at 676 and 1445 cm^−1^; these bands are known to be characteristic bands for methanethiol (CH_3_SH) (Figure S18, Supporting Information).^[^ ^80^ ^]^ Methanethiol as one of the primary VOCs emitted from strawberry fruits, and its concentration is reported to increase during the overripe stage of maturation.^[^ ^81^, ^82^, ^83^ ^]^ After the fungal infection, the SERS intensity of methanethiol significantly increased in the nanobionic plants that were inoculated with infected strawberries compared to those that were not inoculated (Figure 5b). The nanosensor‐embedded plants exhibited increased concentrations of methanethiol released from the infected strawberries starting in 3 days after fungal infection. However, similar to uninfected fruits, the strawberries appeared completely fresh even 4 days after infection when kept in the refrigerator (Figure 5a). Therefore, the sensitive detection of VOC using a nanobionic sensor plant enabled accurate monitoring of fruit decomposition earlier than vision or smell.

Next, monitoring for hazardous volatile chemicals in the air is demonstrated by detecting airborne thiram. When the nanobionic sensor plant was placed with thiram *C_ambient_* of 10 ppb for 60 min, the characteristic SERS bands of thiram appeared at 557, 1143, and 1380 cm^−1^ in the sensor plant (Figure 5c); these bands are identical to the SERS spectrum of 100 µm aqueous thiram solution as well as the SERS spectrum collected from the nanobionic sensor plant infiltrated with 100 µm thiram solution. The nanobionic sensor plant detected airborne thiram *C_ambient_* of 1 ppb (Figure 5d). Many studies have been performed on thiram detection using various nanostructures with high sensitivity,^[^ ^84^, ^85^ ^]^ but mostly have detected thiram in the liquid or solid phase. To the best of our knowledge, airborne thiram detection has not been reported yet, likely because the gaseous thiram concentration conditions required for detection are quite limited due to its low vapor pressure.

Also, the nanobionic sensor plant monitors phenethylamine (PEA) to demonstrate its potential for detecting a drug. PEA has a greater affinity for AgNS@PAA than AgNS@PDDA or AgNS@PVA (Figure S17b, Supporting Information), due to the electrostatic interaction between the amine groups of PEA and the carboxylic acid groups of PAA.^[^ ^86^ ^]^ When the AgNS@PAA‐embedded plant was placed next to the ethanolic PEA solution in the closed chamber; the characteristic SERS bands of PEA were detected at 621, 1000, and 1030 cm^−1^ in the sensor plant; these bands were identical to PEA solution (Figure 5e). At 1 ppm or higher *C_ambient_*, PEA was detected by the nanobionic VOC sensor plants (Figure 5f), and the electrostatic attraction of AgNS@PAA likely contributed to this sensitive detection of PEA containing primary amines. These results could be replicated for the detection of other volatile chemicals that share conformational similarity with PEA. Since certain illicit drugs, such as heroin^[^ ^87^ ^]^ and fentanyl,^[^ ^88^ ^]^ have volatile aromatic components, the applications of nanobionic sensor plants can be further extended to the detection of a variety of regulated substances.

## 3 Conclusion

We have demonstrated VOC‐mediated plant intercommunication using plant nanobionic approaches. A wild‐type living plant turned into the autonomous sensor plant by interfacing with SERS‐active nanosensors, which maintained the plant's unique ability to capture VOCs and facilitate the detection within plant leaves. This nanobionic approach offers a sophisticated analytical tool to study VOCs emitted by plants, allowing for non‐destructive sampling and real‐time monitoring of VOC‐mediated communication between plants, without altering their natural responses to external stimuli. By utilizing the gas‐exchanging process occurring through the stomatal pores during transpiration and leveraging the inherent advantages of plants, such as increased solubility of VOCs, the nanobionic sensor plant showed that plants serve as a VOC concentrator for the communication with neighboring plants. Compared to existing VOC sensors, the nanobionic sensor plants had remarkable reliability and sensitivity and could detect airborne analytes at the ppt level without the need for additional VOC collecting devices or power sources. Furthermore, the versatility of the nanobionic sensor plant is shown through its multiplex detection capabilities owing to the molecular fingerprint characteristics of SERS spectrum. The range of detectable molecules can be expanded by substituting the wrapping polymers on the nanosensor surface that can interact with the target analytes. The label‐free detection methods used in this study can identify the chemical structures of different substances through characteristic SERS band analysis. In addition, the sensor plant could be applied to detect fresh food spoilage at an early stage or monitor hazardous airborne chemicals. The nanobionic sensor plant also has the potential to be used in field applications by its combination with a customized portable Raman device. The nanobionic sensor plant is an innovative tool for investigating plant intercommunications. Furthermore, by harnessing the plant's natural sensor configuration, we envision this nanobionic VOC sensor plant as a compelling sensing platform for various applications, with potential benefits such as improved food safety, enhanced environmental monitoring, and more efficient postharvest management.

## 4 Experimental Section

### Materials

Tetraethyl orthosilicate, ammonium hydroxide (28–30% aqueous solution), 3‐mercaptopropyltrimethoxysilane (3‐MPTS), (3‐aminopropyl)triethoxysilane, ethylene glycol, hexadecylamine, PDDA (very low molecular weight, 35% in H_2_O), PVA (M.W. 89000–98000, 99% hydrolyzed), PAA (average M.W. 100 000, 35% in H_2_O), silver nitrate (> 99.9999% of trace metal basis), 2‐(*N*‐morpholino)ethanesulfonic acid (MES) hydrate, hexadecylamine, 2‐CBT, 4‐FBT, 2,3,5,6‐TFB, thiram, PEA, PEITC, and nicotine were purchased from Sigma–Aldrich (USA). Ethanol (EtOH, absolute 99.9%) and magnesium sulfate (MgSO_4_) were purchased from Daejung (South Korea). All the reagents were used without further purification, and deionized water was used for all the experiments.

### Instruments

The sizes and morphologies of the nanoparticles were observed via transmission electron microscopy (TEM, LIBRA 120, Carl Zeiss, Germany. The conjugation of nanoparticles on the substrate was observed with scanning electron microscopy (SEM, SUPRA 55VP, Carl Zeiss, Germany). The extinction spectra of the nanoparticles were measured with a UV–vis spectrophotometer (Evolution One Plus, Thermo Fisher, USA), and the size and zeta potential were measured using a dynamic light scattering and zeta potential analyzer (Zetasizer Lab, Malvern Panalytical, UK). The SERS spectra were obtained using confocal Raman spectrometers (LabRAM 200VN, Horiba, Japan and XperRAM, Nanobase, South Korea) with 660 and 785 nm lasers and fiber optics‐based customized portable Raman spectrometers with 785 nm laser (Nanobase, South Korea).

### Plant Growth

Commercially available white clover (*Trifolium repens*), watercress (*Nasturtium officinale*), and tobacco (*Nicotiana tabacum*) were seeded in horticultural soil‐filled seedling trays. All plants were grown in a plant growth chamber (HB‐303DH‐O, Hanbaek Scientific, South Korea) for reliable growth control. The plant growth chamber was set at 23 °C, 70% relative humidity, and a 24‐h photoperiod cycle (16‐h light and 8‐h darkness, light intensity 10 mW cm^−2^). Plants were grown for different periods according to experimental usage. White clover and watercress were not used as nanobionic plants until 3–4 weeks after seeding. For plant fluid extraction, white clover and watercress grown from seedlings over a 4‐week period were used. Cultivated tobacco used for the emission of nicotine was grown for more than 4 weeks.

### Synthesis of Polymer‐Wrapped AgNSs

The first step in AgNS preparation was the synthesis of a silica dielectric core using the Stöber method. A portion of 1.6 mL tetraethyl orthosilicate was dissolved in 40.0 mL EtOH, and 3.5 mL ammonium hydroxide solution was added. This reaction mixture was vigorously stirred at 1.4 rcf for 20 h, and then 150 nm silica nanospheres with an average diameter were obtained. Next, the as‐prepared silica nanospheres were washed with EtOH to remove excess reagents. Then, 50 µL of 3‐MPTS and 10 µL of ammonia hydroxide solution were added to the silica nanospheres to functionalize their surfaces with thiol groups to grow AgNSs. The reaction continued overnight, and then the nanoparticles were washed with EtOH to remove excess reagents. Next, 30 mg of silver nitrate (AgNO_3_) was dissolved in 50 mL of ethylene glycol and then added dropwise to 60 µL of thiol‐functionalized silica nanospheres (50 mg mL^−1^). Afterward, the reducing agent hexadecylamine (0.603 g) was added to obtain bumpy AgNSs in 1 h. After the AgNSs were washed with EtOH several times to remove excess reagents, the bumpy AgNSs were functionalized with the PDDA polymer to prepare AgNS@PDDA. AgNSs were dispersed in 30 mL of 0.05 v/v% PDDA aqueous solution, stirred at 1.4 rcf for 1 h, and washed with EtOH multiple times. All the above processes were carried out at room temperature. For the synthesis of AgNS@PVA and AgNS@PAA, a process similar to that of AgNS@PDDA was followed but with some key differences. After the 3‐MPTS treatment, 30 mg of silver nitrate was dissolved in 50 mL of ethylene glycol, and this solution was added dropwise to 60 µL of thiol‐functionalized silica nanospheres (50 mg mL^−1^). The reducing agent hexadecylamine (0.603 g) was then added. For the synthesis of AgNS@PVA, 15 mg of PVA was introduced into the reaction solution. After 1 h of reaction, the solution was washed with EtOH several times. The same procedure was followed for the synthesis of AgNS@PAA but used the PAA polymer (15 mg) instead of PVA.

### Demonstration of the Biocompatibility of Polymer‐Wrapped AgNSs

To demonstrate the biocompatibility of the nanoparticles, 0.1 mg mL^−1^ AgNS@PDDA, AgNS@PVA, and AgNS@PAA were suspended in 5 mm MES–MgSO_4_ buffer (pH 5.7) and the colloidal mixtures were infiltrated into the abaxial side of white clover leaves after 3–4 weeks of growth. The chlorophyll content of the three groups was measured for 4 weeks at 3‐to‐4‐day intervals using a SPAD meter (502 Plus, Minolta, Japan). The chlorophyll content of each group was measured using six individual leaves from different plants. Additionally, to assess the biocompatibility at the cellular level, a propidium iodide assay was performed. The nanoparticles and buffer‐embedded leaves were cut into 5 mm wide leaf discs after 1 h after the infiltration. The leaf discs were submerged in 50 mg L^−1^ propidium iodide solution for 30 min. Then, the stained discs were transferred to perfluorodecalin‐filled polydimethylsiloxane (PDMS, Carolina Observation Gel, USA) well on glass slides. The glass slide was sealed up with cover glass, and the epidermis layer of the samples was imaged by a 40× water immersion objective of confocal fluorescence microscope (CLSM SP8 X, Leica, Germany). Propidium iodide molecules penetrate apoptotic or dying cell membrane and stain nucleic acids, but do not penetrate viable cells. Therefore, cells stained their DNA were considered to undergo cell death or other adverse effect upon infiltration of the nanosensor. The ratio of dead cells was evaluated based on twenty‐four images (290  ×  290 µm) taken from four different plants.

### Plant Physiological Responses to AgNS@PDDA Infiltration

The impact of AgNS@PDDA infiltration on plant physiology was assessed by quantifying the net CO_2_ assimilation rate (A_net_), evapotranspiration (E_T_), and stomatal conductance to water vapor (g_sw_) from gas exchange and chlorophyll fluorescence measurements at the leaf scale. Ten pots of clover plants were prepared and incubated for 21 days until the third trifoliate fully expanded. To ensure reliable measurements, the plants were moved to an indoor greenhouse, which provided a stable environmental condition at 25 °C, a relative humidity of 60%, and a photosynthetic photon flux density of 200 µmol m^−2^ s^−1^. After the plants were acclimatized to the greenhouse conditions for 30 min, AgNS@PDDA was infiltrated into plant leaves in randomly selected pots. Gas exchange measurements were conducted for the AgNS@PDDA‐embedded pots (*N* = 6) and control pots (N = 6) in the greenhouse at 10 min before infiltration and 60, 70, 90, 120, and 180 min after the infiltration by using a portable photosynthesis system (Li‐6800, LI‐COR Inc., USA). To minimize the impact of diurnal variation in plant physiology on the measurements, infiltration was always conducted at the same time of day. The instrument's leaf chamber clamped on and measured one trifoliate at a time. The CO_2_ concentration was set to 400 µmol mol^−1^, and other environmental conditions of the chamber were identical to those of the greenhouse environment. Each measurement was conducted over a 6 cm^2^ area, covering all three leaflets of the trifoliate compound leaves, with AgNS@PDDA nanosensors infiltrated into the individual leaflets (~0.13 cm^2^). The stabilization criteria for the initiation of the logging process included temporal slopes of ΔH_2_O and ΔCO_2_ less than 0.1 and 0.5 µmol m^−2^ s^−1^, respectively. Due to the difference in the leaf area between the sample leaves, all data were corrected for the corresponding total leaf area, which was quantified by analyzing the digital camera images.

### Detection of Airborne VOCs Using a Nanobionic Sensor Plant

A colloidal suspension of polymer‐wrapped AgNS (AgNS@PDDA, AgNS@PVA, or AgNS@PAA) in 5 mm MES‐MgSO_4_ buffer (pH 5.7) was infiltrated into the plant leaf by using a needleless 1 mL syringe. During infiltration, low pressure was applied to the leaf to ensure that no mechanical damage occurred. Infiltration was carried out until the periphery of the infiltration point was sufficiently soaked by the polymer‐wrapped AgNS suspension. The nanoparticle‐infiltrated leaf was rinsed with water to remove the remaining nanoparticles from the surface. The nanosensor‐infiltrated plant was ready for use as a sensor plant after at least 1 h of storage to allow the remaining buffer to dry. A controlled environment was crucial for the exposure of the nanobionic sensor plant to chemicals. To achieve this, an acrylic chamber with an inner volume of 125 L (50 × 50 × 50 cm) was prepared. The nanobionic sensor plant was positioned on one corner of the acrylic chamber, while the Petri dish for the analyte was placed on the opposite corner, 70 cm away from the nanobionic sensor plant. A precise amount of EtOH solution containing the analyte chemical at the desired *C_ambient_* was added onto a petri dish. The acrylic chamber was immediately sealed after the analyte exposure, ensuring that the nanobionic sensor plant was exposed to the analyte from the moment the EtOH solution on the dish evaporated. All experiments, except for the time‐dependent incubation experiments, were carried out with a 60‐min exposure to VOCs. The nanosensor‐embedded leaf was placed on a glass slide under the microscope while remaining attached to the living plant for SERS measurement. All measurements of the nanobionic sensor plants were carried out with a confocal Raman spectrometer, with 785 nm photoexcitation. The acquisition was performed with a 40× objective lens (0.75 NA), and the incident laser power applied to the sample was set as 2 mW, which the previous study had shown does not cause significant stress or damage to plant cells.^[^ ^31^ ^]^ This laser irradiation setup did not cause significant changes in chlorophyll contents and cellular stress in the nanobionic sensor plants (Figure S19, Supporting Information). SERS intensity maps were obtained in point‐by‐point mapping acquisition mode, in which the intervals between the points were 3 µm on both the x‐ and y‐axes, and the acquisition time was 100 ms for each point. For the construction of false‐color maps of SERS intensity, the mapping images were arranged according to the intensities of the Ag···N stretching band at 235 cm^−1^ and 4‐FBT bands at 1076 cm^−1^ after baseline subtraction.

### In Vitro SERS Measurement on the SERS Substrate

The process of preparing the in vitro SERS substrate involved several steps. First, a two‐inch silicon wafer was cleaned with water and acetone, and then the wafer was placed in piranha solution to oxidize the surface and eliminate any organic residue. Once the surface was hydroxylated, the sample was placed into a solution of 0.02 mm poly(4‐vinylpyridine) (P4VP) dissolved in EtOH for 1 h. After the reaction was complete, any unreacted P4VP was washed away with EtOH. Next, the wafer was reversely laid into a gently stirring suspension of AgNS@PDDA at 0.05 rcf for another hour. Again, any unreacted AgNS@PDDA was washed away with EtOH. Once the process was complete, the prepared in vitro SERS substrate was dried. To mimic the aqueous environment of a nanobionic sensor plant, 180 µL of distilled water was added onto the surface to cover the in vitro SERS substrate. After incubation with airborne 4‐FBT, SERS measurements were carried out using a confocal Raman spectrometer with 2 mW laser power and a 5 s acquisition time. For both types of in vitro SERS substrates, measurements were performed using a 100× objective lens (0.60 NA).

### In Vitro SERS Measurement in Solution

Polymer‐wrapped AgNS were dispersed in DI water at 0.1 mg mL^−1^ nanoparticle concentration. The ethanolic solution of the analytes was mixed with the nanoparticle suspension, followed by 1 h of vigorous mixing. After mixing, SERS measurements of the solution in a glass capillary tube were carried out with a confocal Raman spectrometer. A 660 nm laser was used as the excitation source for measurement, with an incident 2.5 mW sample power and a 60 s acquisition time.

### Detection of VOCs from Infected Strawberry Fruits

The *B. cinerea* strain B05.10 was cultured on potato dextrose agar at 25 °C under near‐UV light (wavelength, 352 nm; Sankyo Denki, Japan). After 10 days, the conidia were harvested with sterile distilled water, filtered through Miracloth (Millipore, USA), and adjusted to a concentration of 10^6^ spores mL^−1^.

Strawberry (*Fragaria x ananassa*, cultivar “Seolhyang”) was used for the infection assay. Strawberries were surface‐sterilized with 1% NaOCl, rinsed three times with sterile distilled water, and air‐dried for 5 min. Conidia suspensions (1 mL) were sprayed onto the surfaces of the strawberries, and sterile distilled water was used as a control. The sprayed strawberry samples were placed in a plastic chamber and stored at 4 °C. After 24, 48, 72, and 96 h of storage, the strawberries were placed at room temperature for analysis. For each experimental set, 4 strawberry fruits were removed from storage and incubated in the acrylic chamber with a nanobionic sensor plant for 1 h.

### Detection of Plant Wound Signaling VOCs

Three herb plants, coriander (*Coriandrum sativum*), rose geranium (*Pelargonium graveolens*), and applemint (*Mentha Suaveolens*), were purchased from the local flower market. For each plant, 4–5 leaves were cut one‐third from the edge, and another ten leaves were scratched with a knife. The wounded herb plants were then placed in an acrylic chamber with a nanobionic sensor plant for 2 h (*n* = 2).

### Monitoring VOCs Using a Portable Raman Spectrometer

The portable Raman spectrometer used fiber‐based optics, was equipped with a 10× objective lens and was customized for signal acquisition during incubation. The spectrometer was used in conjunction with a clover leaf embedded with AgNS@PDDA, with the measurement unit of the spectrometer and the clover plant placed in an acrylic chamber. As the nanosensor‐embedded leaf was placed in front of the measurement unit by a rubber clip while remaining attached to the living plants, the nanobionic sensor plant was incubated with 4‐FBT *C_ambient_* of 1 ppm. During the incubation, a 785 nm laser with a 7.5 mW incident power irradiated the clover leaf to enable Raman spectrum measurements. The SERS signals were collected from the nanobionic sensor plant every 12 s for 1 h, with an acquisition time of 1 s. To simultaneously monitor multiple VOCs with the portable Raman device, the nanobionic sensor plant was placed in an acrylic chamber containing 4‐FBT *C_ambient_* of 5 ppm, 2‐CBT *C_ambient_* of 5 ppm, and 2,3,5,6‐TFB *C_ambient_* of 2 ppm. Each VOC was separately placed in the box as an ethanolic solution and then eventually vaporized to reach the desired *C_ambient_* of the gaseous mixture in a few minutes. The SERS signals were acquired from the nanobionic sensor plant using the same methods for single‐type VOC monitoring.

## Conflict of Interest

The authors declare no conflict of interest.

## Author Contributions

Y.S.C. and W.K.S. contributed equally to this work. S.Y.K. and D.H.J. conceived the experiments, obtained funding, and oversaw the research. Y.S.C. and W.K.S. synthesized and characterized the nanoparticles and performed sensing experiments. H.K., D.S., and M.G.K. assisted with the experiments. J.P. and H.S. prepared the infected strawberry models. S.C. and H.K. measured stomata conductance. Y.S.C., W.K.S., S.Y.K., and D.H.J. analyzed the data. Y.S.C., S.Y.K., and W.K.S. wrote the initial manuscript, edited by S.Y.K. and D.H.J.

## Supporting information



Supporting Information

## Data Availability

The data that support the findings of this study are available from the corresponding author upon reasonable request.
