# Supplementary material for: Real‐Time Monitoring of Volatile Organic Compound‐Mediated Plant Intercommunication Using Surface‐Enhanced Raman Scattering Nanosensor
Source: Adv Sci (Weinh). 2024 Dec 24;12(7):2412732. doi: 10.1002/advs.202412732 (PMC11831534; doi:10.1002/advs.202412732)
Supplement: Supplementary file 1 — Supporting Information [file ADVS-12-2412732-s001.pdf]

ADVANCED SCIENCE
Open Access

Supporting Information

for *Adv. Sci.*, DOI 10.1002/advs.202412732

Real-Time Monitoring of Volatile Organic Compound-Mediated Plant Intercommunication Using Surface-Enhanced Raman Scattering Nanosensor

Yun Sik Choi, Won Ki Son, Hyuna Kwak, Jiyeun Park, Sumin Choi, Daeseob Sim, Min Gyeong Kim, Hyungsuk Kimm, Hokyoung Son, Dae Hong Jeong and Seon-Yeong Kwak**

Supporting Information

Real-time monitoring of volatile organic compound-mediated plant intercommunication using surface-enhanced Raman scattering nanosensor

Yun Sik Choi, Won Ki Son, Hyuna Kwak, Jiyeun Park, Su Min Choi, Dae Seob Sim, Min Gyeong Kim, Hyungsuk KHimm, Hokyoung Son, Dae Hong Jeong, Seon-Yeong Kwak**

1. Calculation of stomatal conductance

The resistance that gas molecules undergo when it passes through stomata is expressed as 'stomatal resistance (r_s)' or its reciprocal form, 'stomatal conductance (g_s)'.

Stomata conductance can be calculated by following equation:[1]

$$g_s = \frac{DmA_s}{(L + 2 \times end\ correction)} \quad (1)$$

where D is the diffusion coefficient [m^2/s] of gas molecules propagating a medium, m is the stomatal density [m^{-2}], A_s is the area [m^2] of stomatal pore and L is the depth [m] of stomata pore.

The diffusion coefficient of 4-FBT in air was not found in literatures, therefore it was calculated theoretically by Chapman-Enskog theory.

$$D_{4-FBT,air} = \frac{1.86 \times 10^{-27} T^{\frac{3}{2}} \sqrt{\frac{1}{M_{4-FBT}} + \frac{1}{M_{air}}}}{p\sigma_{4-FBT,air}\Omega} \quad (2)$$

where T is the absolute temperature [K], M_{4-FBT} and M_{air} are the molar masses [g/mol] of 4-FBT and air, p is the pressure [atm], $\sigma_{4-FBT/air}$ is the average collision diameter [m] between 4-FBT and air, and Ω is a dimensionless temperature dependent collision integral.

The average collision diameter, $\sigma_{4-FBT,air}$ was calculated by:

$$\sigma_{4-FBT,air} = \frac{1}{2}(\sigma_{4-FBT} + \sigma_{air}) \quad (3)$$

where σ is the Lennard-Jones parameter. σ_{air} was 3.711 Å according to previous research.[2] σ_{4-FBT} was estimated as 9.68 Å by our DFT calculation and method used in literature.[3]

The temperature-dependent collision integral, Ω was calculated by approximation:[4]

$$\Omega = \frac{1.06036}{(T^*)^{0.15610}} + \frac{0.19300}{e^{0.47635T^*}} + \frac{1.03587}{e^{1.52996T^*}} + \frac{1.76474}{e^{3.89411T^*}} \quad (4)$$

T^* can be calculated by:

$$T^* = \frac{Tk_B}{\varepsilon_{4-FBT,air}} \tag{5}$$

where k_B is the Boltzmann constant and $\varepsilon_{4-FBT,air}$ is the energy of molecular interaction [J] for the binary system of 4-FBT and air. It is given by:

$$\varepsilon_{4-FBT,air} = \sqrt{\varepsilon_{4-FBT}\varepsilon_{air}} \tag{6}$$

ε_{air}/k_B was 7.86 K according to data previous research[2] and ε_{4-FBT} was calculated by empirical correlation equation:

$$\varepsilon_{4-FBT}/k_B = 1.15T_b \tag{7}$$

where T_b is normal boiling temperature of 4-FBT, 441.15 K.

Finally, $D_{4-FBT,air}$ was calculated as 3.66×10^{-6} m^2/s in room temperature (25 °C).

Under microscopic observation, the stomatal density of clover leaves was estimated to be 132.1×10^6 m^{-2}. Stomatal pore was considered as an ellipse, where major axis (a), stomatal length, remained unchanged, but minor axis (b), which coincides with the stomatal aperture (f), was found to be vary in response to environmental factor or abscisic acid (Figure S6). The depth of the stomatal pore was assumed to be 15 μm. The end correction, a term added to consider that effective diffusion length is longer than actual stoma pore depth, was chosen specifically for its applicability to our study. We opted for Waggoner's correction[5] due to its relevance to our research:

$$end\ correction = b \cdot \ln(4a/b) = f \cdot \ln(4a/f) \tag{8}$$

We calculated the stomatal conductance of clover and tabulated it in Table S1 corresponding to stomatal apertures.

In addition to stomatal conductance, resistance from the leaf boundary layer (LBL) is present when gas molecules enter a plant leaf. When there is no convection and interference, Vesala[6] gives the resistance of the LBL:

$$r_{LBL} = \frac{1}{4mD\sqrt{ab}} \tag{9}$$

where r_{LBL} is the resistance of LBL.

Using the same method as electric resistance, the total resistance (r_{sum}) can be calculated.

$$\frac{1}{r_{sum}} = \frac{1}{r_s} + \frac{1}{r_{LBL}} = g_s + \frac{1}{r_{LBL}} \tag{10}$$

2. Calculation of the VOC concentration inside stomata

The gas exchange in plants, specifically oxygen and carbon dioxide exchange, usually occurs through the stomata via free diffusion.[7] The entry of VOCs in the air into the nanobionic

plant sensor and their reaching the plant fluid depends on their diffusion through stomata pores into the airspace inside the plant leaf. Therefore, the flux of VOCs follows the concentration gradient of VOCs and determines the diffusion rate of VOCs through the stomata pore. Since the infiltrated SERS nanosensors were placed adjacent to the substomatal cavity, the gas influx through the stoma was considered to follow a linear diffusion along the pathway from the ambient air to the inside of the plant, from a narrow domain perspective. We have simplified the structure of a leaf and illustrated it (Figure S7).

From definition of conductance,

$$J \approx -g\Delta C = g(C_{ambient} - C_{plant,air}) \tag{11}$$

where J is the flux [mol/m^{-2}s^{-1}] of VOC.

To calculate the change in concentration inside stomata, we used the following formula:

$$\frac{dC_{plant,air}}{dt} = \frac{1}{V_{plant,air}}\frac{dn_{plant,air}}{dt} = \frac{JA_c}{V_{plant,air}} \approx \frac{A_c}{V_{plant,air}} g_{sum}(C_{ambient} - C_{plant,air}) \tag{12}$$

where A_c is the area of the aerial column that the VOC passes through after the stomatal pore, $n_{plant,air}$ is number of VOC molecules in the airspace inside the leaf, and $V_{plant,air}$ is the volume of airspace inside the plant leaf. We have assumed A_c as constant as elliptical stomata close their pore like 'drawbridge' with their guard cells tilted above the plane of the epidermis[1]

Assuming a constant ambient concentration throughout the process, we can solve the differential equation in the following way:

$$\frac{1}{C_{ambient} - C_{plant,air}} dC_{plant,air} \approx \frac{g_{sum}A_c}{V_{plant,air}} dt \tag{13}$$

$$C_{plant,air}(t) \approx C_{ambient}(1 - \exp\left(-\frac{g_{sum}A_c t}{V_{plant,air}}\right)) \tag{14}$$

3. VOC binding at SERS nanosensor

The VOCs in the gas phase dissolve into the plant fluid according to the dimensionless Henry constant, H_S^{CC}, and subsequently bind to the SERS nanosensors, therefore leading to the generation of SERS signals. The VOC binding processes can be expressed as follows:

$$A(plant, aq) + NP \Leftrightarrow A(bound) \qquad K_{bound} = \frac{C_{bound}}{C_{plant,aq}C_{NP}} \tag{15}$$

K_{bound} is the binding constant of the VOC molecules to the surface of SERS nanosensors in an aqueous solution, C_{bound} is the concentration of VOC-bound nanosensors, and C_{NP} is the concentration of nanosensors without bound VOC. This constant is defined based on the assumption that the adsorption of the VOC molecules on SERS nanosensors follows the Langmuir isotherm model of one-layer adsorption, similar to other molecular adsorption methods reported in previous studies.

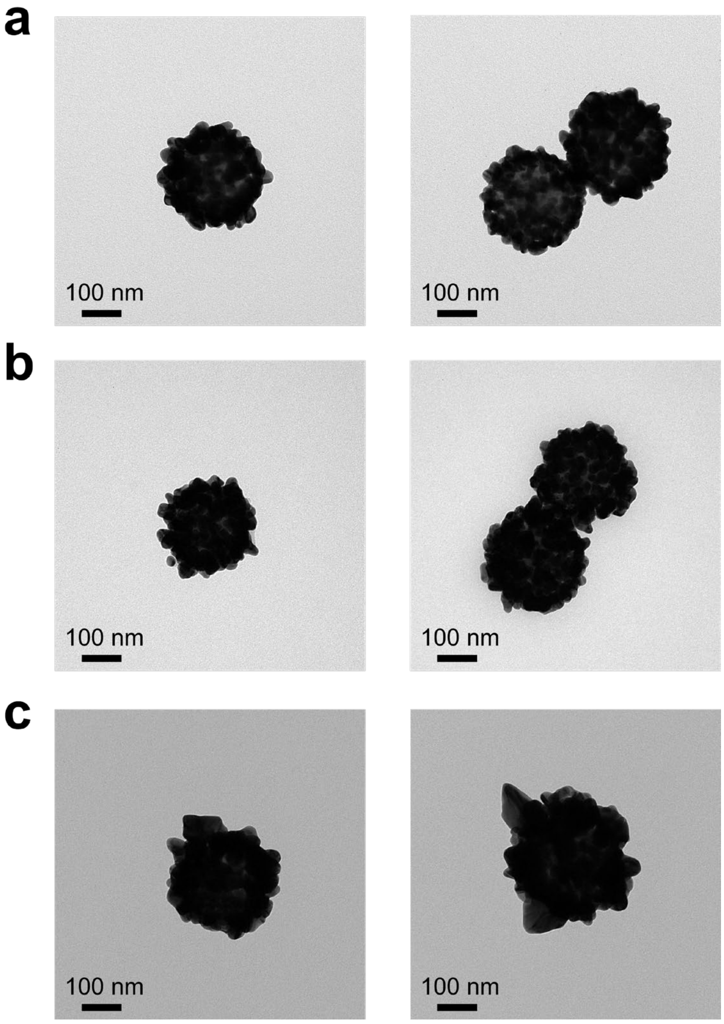

Figure S1. Size and morphology of AgNS@PDDA, AgNS@PVA and AgNS@PAA. Transmission electron microscopy images of a) AgNS@PDDA, b) AgNS@PVA and c) AgNS@PAA. Scale bar is 100 nm.

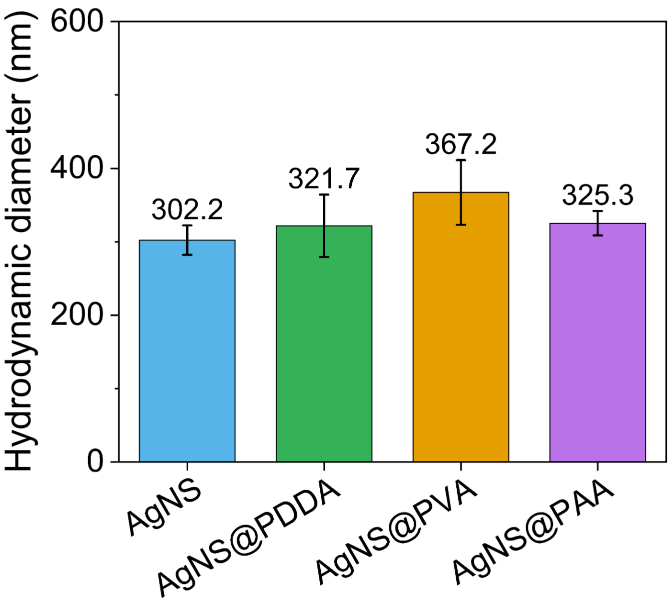

Figure S2. Hydrodynamic diameter of AgNS@PDDA (green), AgNS@PVA (dark yellow), and AgNS@PAA (purple) compared with that of bare AgNSs (blue). The data are represented as mean±s.d. based on six individual experiments at each point.

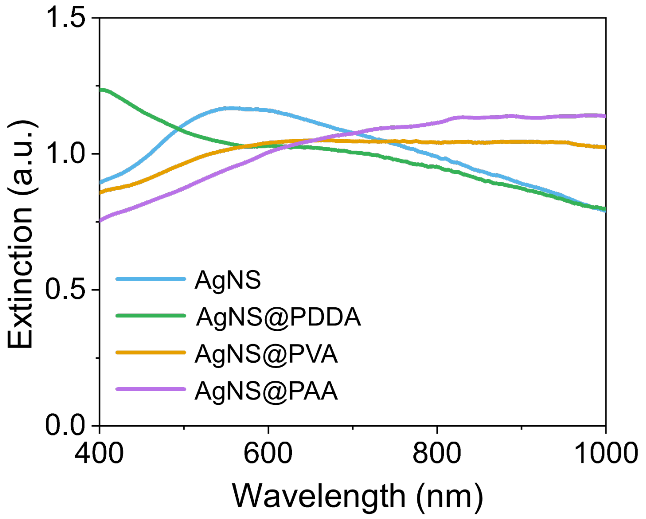

Figure S3. UV–visible extinction spectra of AgNS@PDDA (green), AgNS@PVA (dark yellow), and AgNS@PAA (purple) compared with that of bare AgNSs (blue) as a reference.

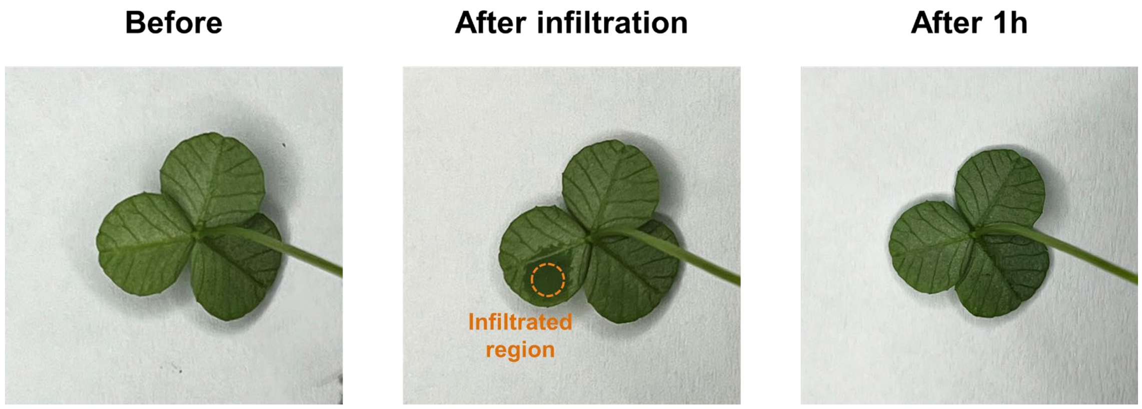

Figure S4. The progress for an hour after infiltration of AgNS@PDDA nanosensor (0.1 mg ml^{-1}) into the clover leaf as attached to the clover plant.

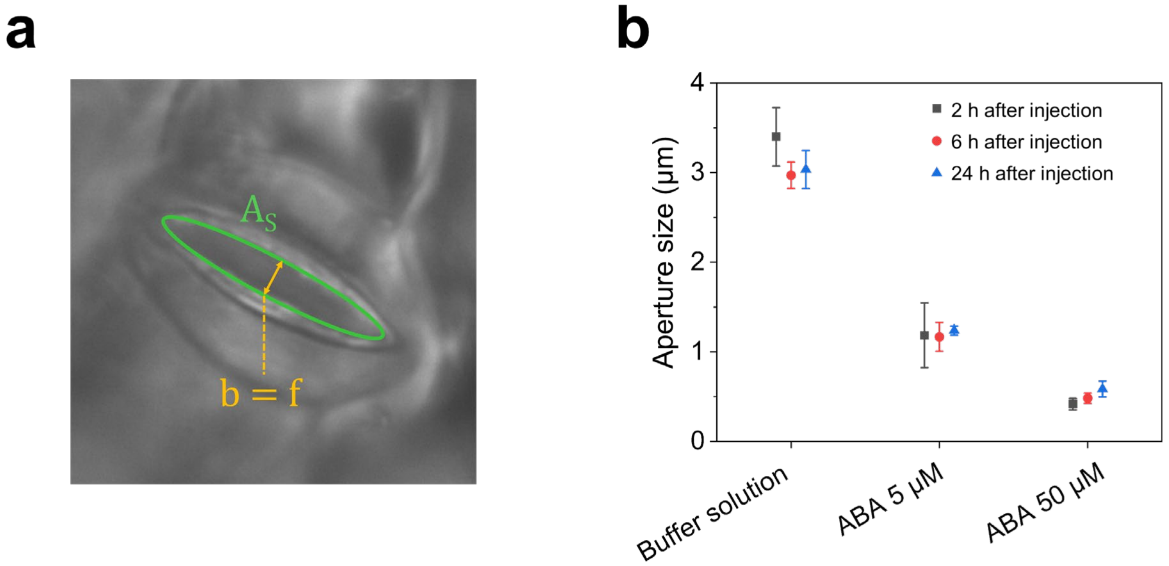

Figure S5. Stomata aperture size of clover with abscisic acid. a) Measuring the size of stomata aperture pore (b=f) at bright field image of clover leaf. b) The aperture size of ABA-treated clover stomata was measured at 2 hours (black), 6 hours (red) and 24 hours (blue) after ABA treatment.

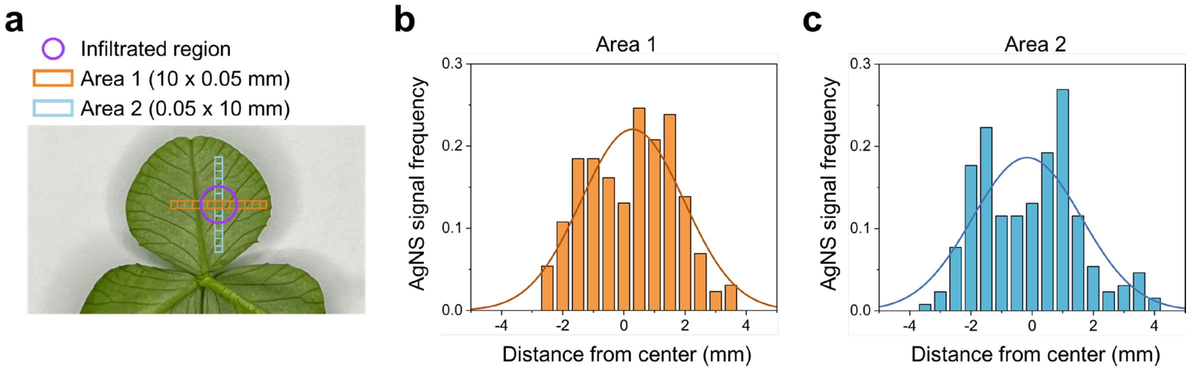

Figure S6. Distribution of AgNS@PDDA nanosensor particles within the plant leaf of nanobionic sensor plant. a) Schematic of Raman measurement conducted on two orthogonal directions. Measured areas had a width of 0.05 mm and a length of 10 mm. Each area was divided by intervals with 0.5 mm length, and Raman mapping measurement was conducted for each interval with 10x objective lens, 785 nm photoexcitation with 2 mW laser power and 1 s light acquisition. b-c) Histograms for the AgNS signal detection frequency in area 1 (b) and area 2 (c). The solid curve in each plot represents the Gaussian fit curve to the histograms.

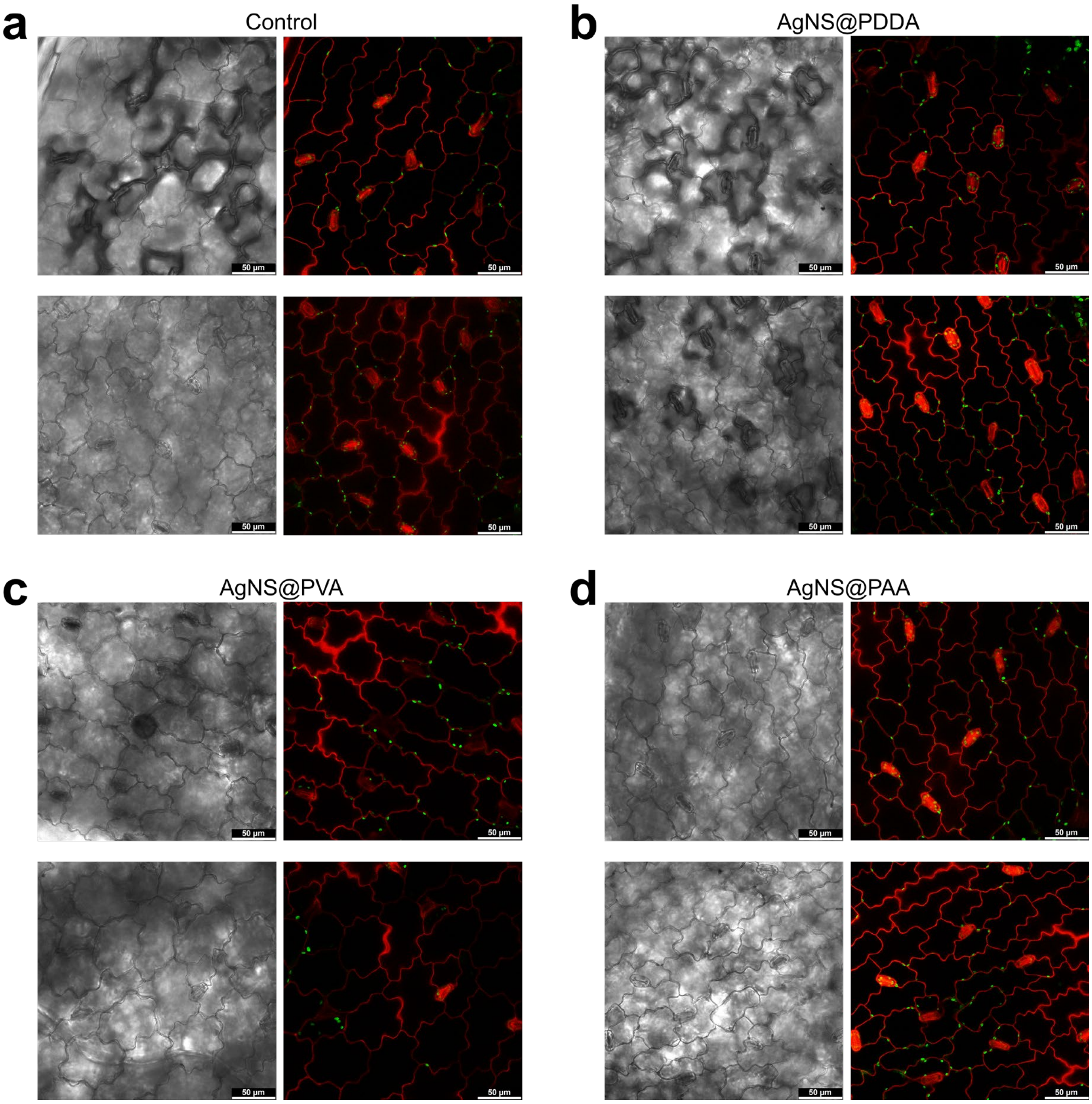

Figure S7. Representative confocal fluorescence micrograph and brightfield images of propidium iodide-stained clover leaves. a) the leaves without nanoparticle infiltration b-d) the leaves embedded with AgNS@PDDA (b), AgNS@PVA (c) and AgNS@PAA (d). AgNS@PDDA, AgNS@PVA, and AgNS@PAA (0.1 mg ml^{-1}) were infiltrated into the abaxial side of white clover leaves. Propidium iodide fluorescence dye appeared as red, while chlorophyll auto-fluorescence appears as green. Scale bar is 50 μm.

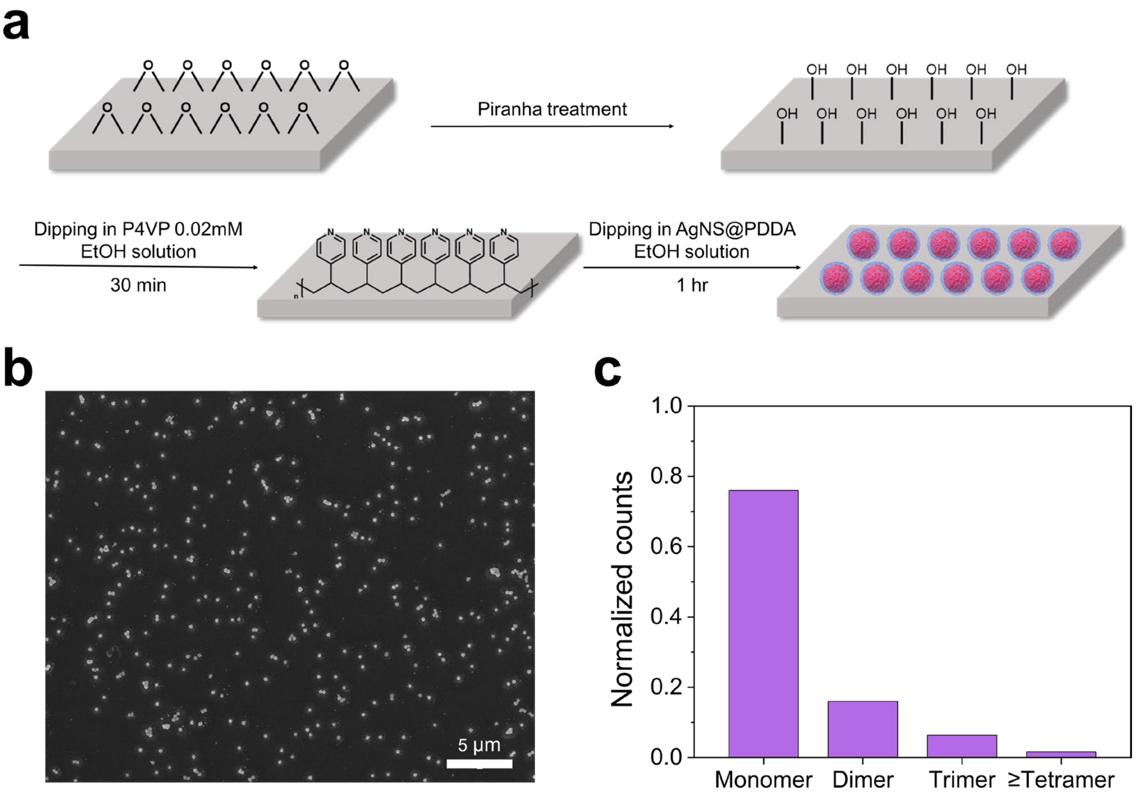

Figure S8. Preparation and images of wafer-based SERS substrate. a) Synthetic scheme of wafer-based SERS substrate on silicon wafer. b) Scanning electron microscope image of wafer-based SERS substrate. Scale bar is 5 μm. c) Normalized counts of AgNS@PDDA in forms of monomers, dimers, trimers and others, quantified from scanning electron microscope images of a wafer-based SERS substrate (n=487).

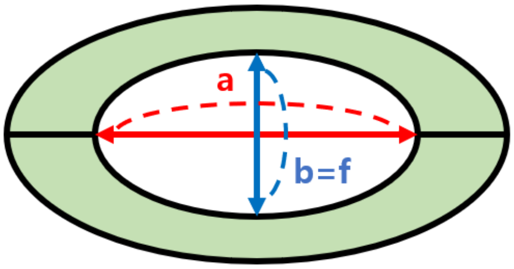

$$A_S = \frac{ab}{4\pi} = \frac{af}{4\pi}$$

Figure S9. Area of stomata pore. The stomatal pore is generally elliptical, with a major axis (a) and a minor axis (b).

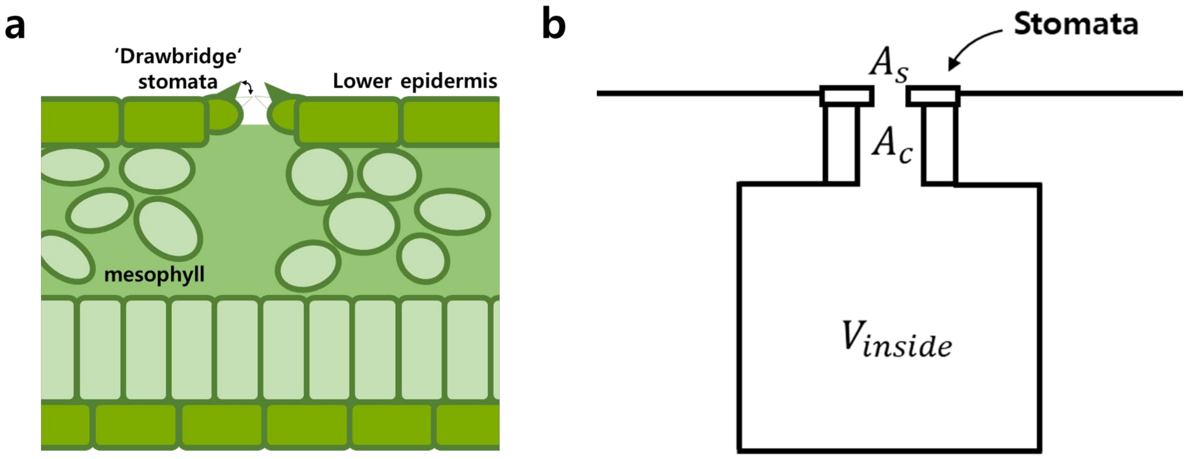

Figure S10. Schematic illustration of diffusion pathway through stomata. a) An illustration depicts the cross-section of a plant leaf featuring 'drawbridge stomata,' a type of stomatal opening that resembles a drawbridge. b) A simplified schematic structure derived from (a). The space in the mesophyll beneath the stomata is represented as a simple box, designated as the $\boldsymbol{V_{inside}}$, and the passage between the stomata and the plant's interior is kept constant. The area of the stomata, however, varies with the treatment of ABA.

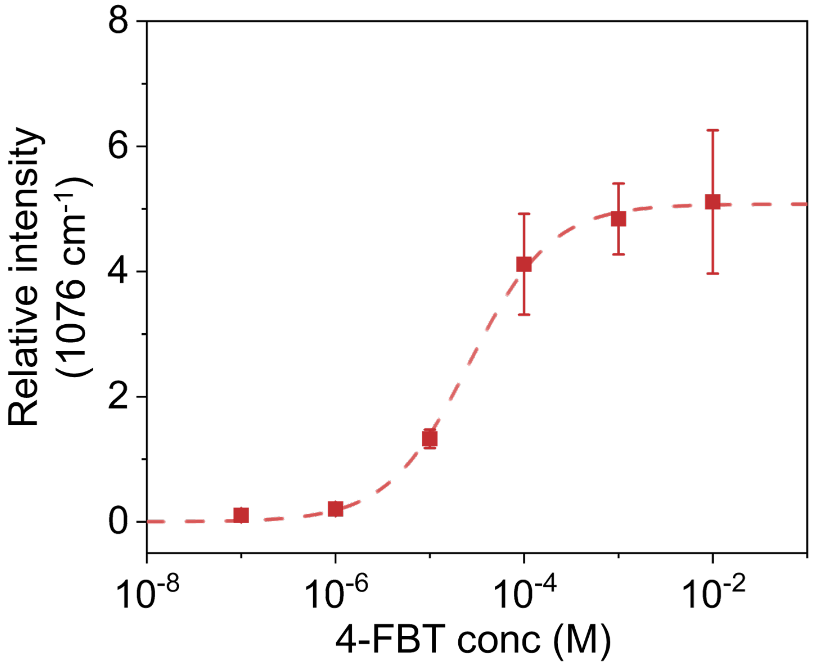

Figure S11. Calibration curve of aqueous 4-FBT concentration versus relative SERS intensity. Dashed line represents statistical fitting according to Langmuir isotherm model (q_{max}=5.03 ± 0.18 and K_L = 3.68 × 10^4 ± 0.82 × 10^4).

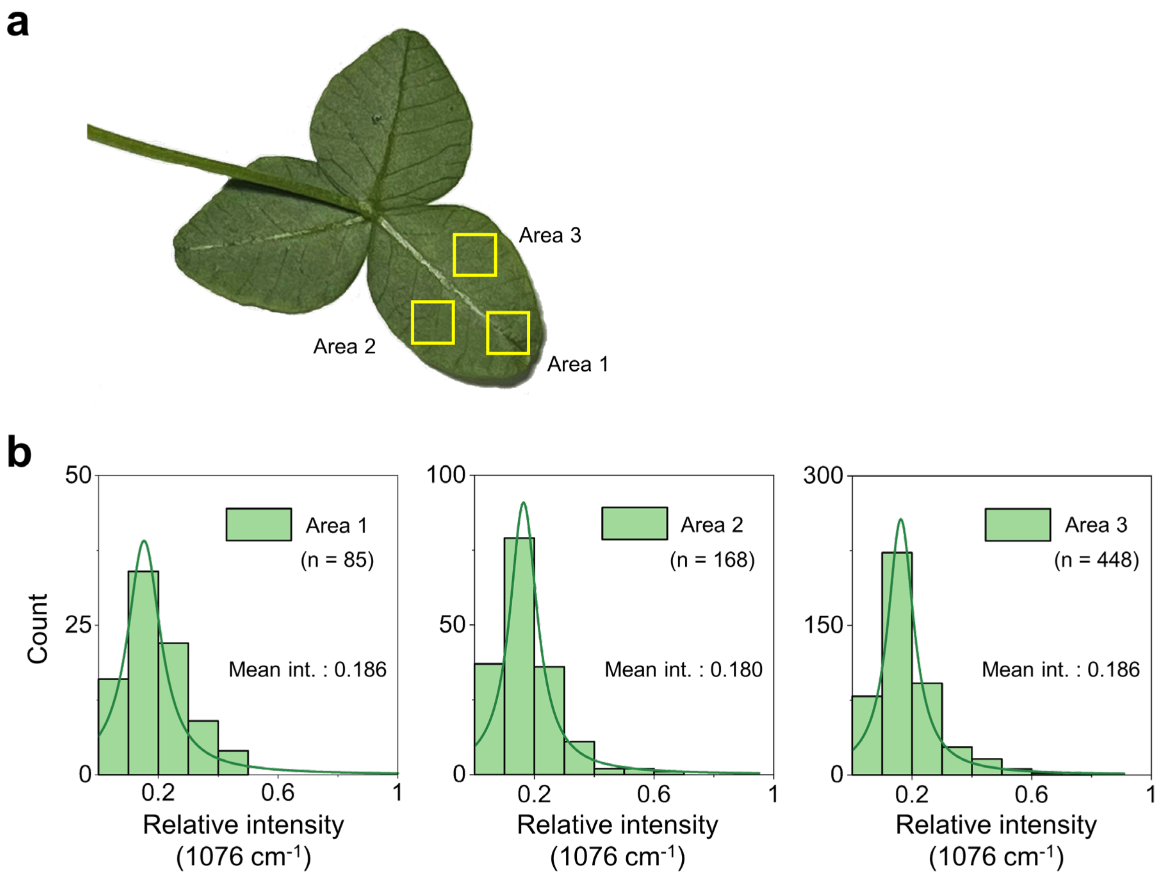

Figure S12. Consistency of VOC SERS signal on nanobionic sensor plant. a) Photograph of a clover leaf used for SERS measurement after 4-FBT exposure. b) Representative histograms for the relative intensities of 4-FBT (1076 cm^{-1}) in Raman mapping measurements for areas 1 to 3 at (a), after exposed to 4-FBT with $C_{ambient}$ of 1 ppb. The solid curve in each plot is the curve fitted to the histograms.

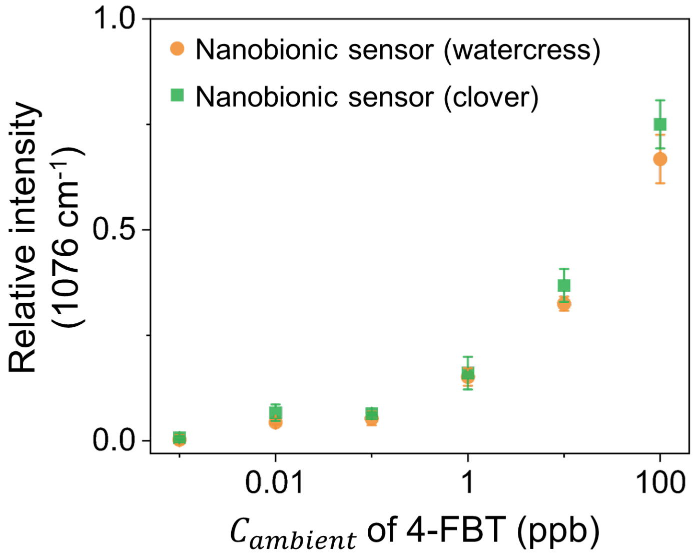

Figure S13. Comparison of the SERS signal intensity at 1076 cm^{-1} for 4-FBT between the signals detected from nanosensor-embedded clover and nanosensor-embedded watercress. The SERS intensities were normalized to the intensity of the AgNS band at 235 cm^{-1} and are expressed as the relative intensity. The data are represented as mean±s.d. based on three individual experiments at each point (N=3).

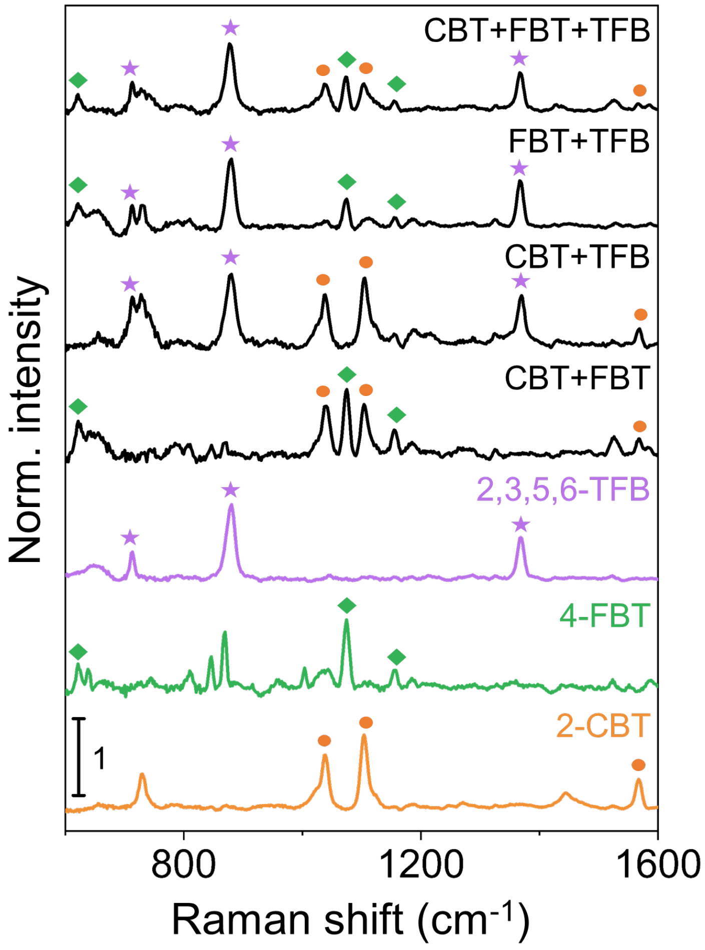

Figure S14. Multiplex VOC detection on nanobionic sensor plant utilizing the customized portable Raman device. SERS spectra were measured at nanobionic sensor plant after incubated with either multiple VOCs or their gaseous mixtures. SERS spectra of VOCs, namely, 0.1 ppm of 2-CBT (orange), 0.1 ppm of 4-FBT (green), 0.1 ppm of 2,3,5,6-TFB (purple) and their gaseous mixture, respectively 2-CBT+4-FBT, 2-CBT+2,3,5,6-TFB, 4-FBT+2,3,5,6-TFB and 2-CBT+4-FBT+2,3,5,6-TFB (black). The $\boldsymbol{C_{ambient}}$ of each molecule was 0.5 ppm for 2-CBT, 0.5 ppm for 4-FBT, and 0.2 ppm for 2,3,5,6-TFB in the mixture. The symbols mark the characteristic bands contributed by 2-CBT (orange circle), 4-FBT (green square), 2,3,5,6-TFB (purple star). SERS measurement was conducted with portable Raman device equipped with a near-IR (785 nm) aser, obtained with 30 s of acquisition time and 17 mW of laser power.

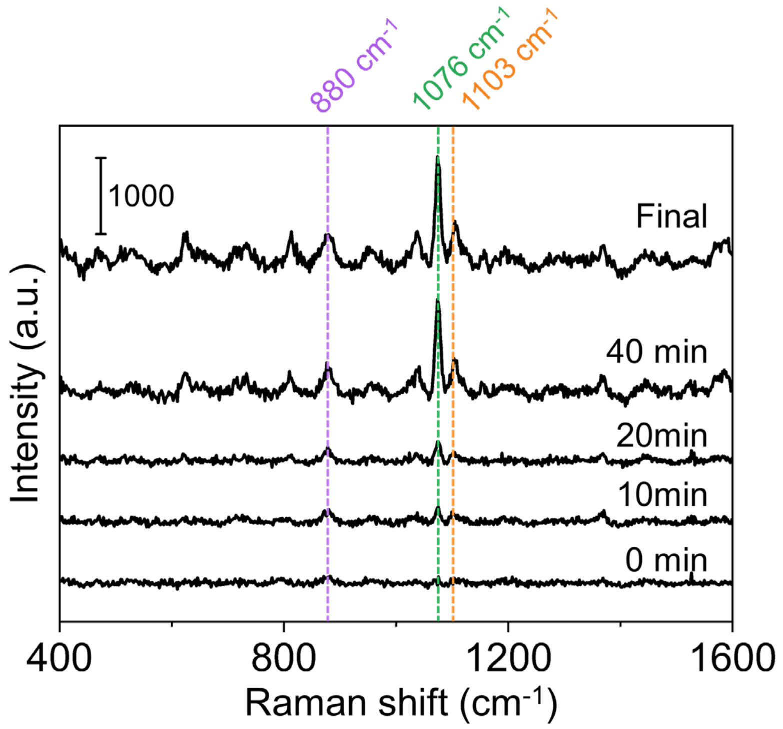

Figure S15. Changes in the observed SERS spectrum over time during continuous monitoring of multiple VOCs. Real-time change of SERS signal from nanobionic sensor plant, measured during incubation with gaseous mixture of 2-CBT, 4-FBT, and 2,3,5,6-TFB. The $\boldsymbol{C_{ambient}}$ of each molecule was 5 ppm for 2-CBT, 5 ppm for 4-FBT, and 2 ppm for 2,3,5,6-TFB in the mixture. Dashed lines are corresponding to characteristic bands for each VOC, respectively 1103 cm^{-1} for 2-CBT (orange), 1076 cm^{-1} for 4-FBT (green), and 880 cm^{-1} for 2,3,5,6-TFB (purple). SERS measurement was conducted with portable Raman device equipped with ×10 objective lens and a near-IR (785 nm) laser, obtained with 1 s of acquisition time and 7.5 mW of laser power.

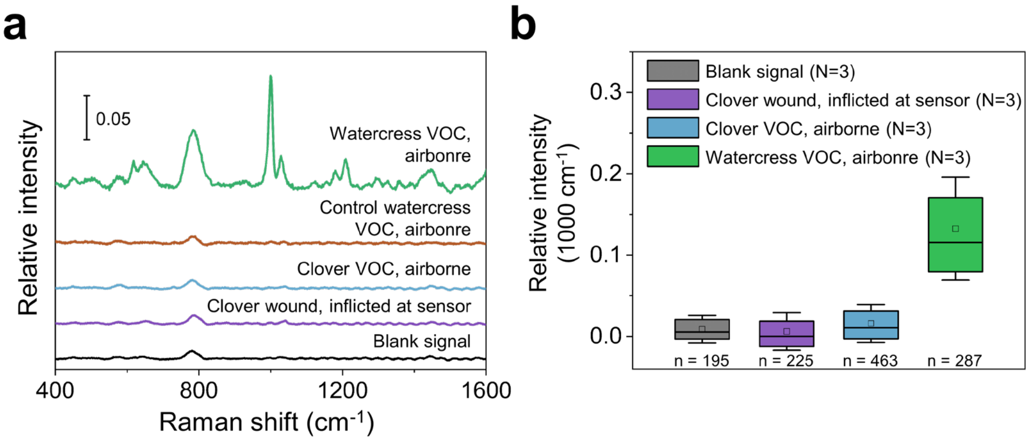

Figure S16. Comparing SERS signals of watercress VOC with SERS signals from other plant wounds. a) Representative SERS signals were obtained from nanobionic sensor plant after incubation with wounded watercress (green) or control watercress without wound (brown). Then, SERS signals were measured at nanobionic sensor plant, after wounds were inflicted at nanobionic sensor plant itself (purple) or incubated with the other wounded clover (blue). Obtained signals were compared with normal background signal from nanobionic sensor plant (black). b) SERS signal intensity comparison between control watercress without wound (gray), clover wound inflicted at nanobionic sensor plant itself (purple), volatile from clover with wound (blue) and volatile from watercress with wound (green). The intensities of bands 1000 cm^{-1}, which assigned to PEITC, were normalized by the intensity at 235 cm^{-1} corresponding to the AgNS band of the nanoparticle. SERS intensities were measured in three individual experiments for each group. SERS signal intensities were represented as a box plot where the box ranges from the first to third interquartile with the horizontal line and small rectangular indicating median and mean, respectively, and whisker indicating ±1× standard deviation. The number of spectra in each group was represented as n.

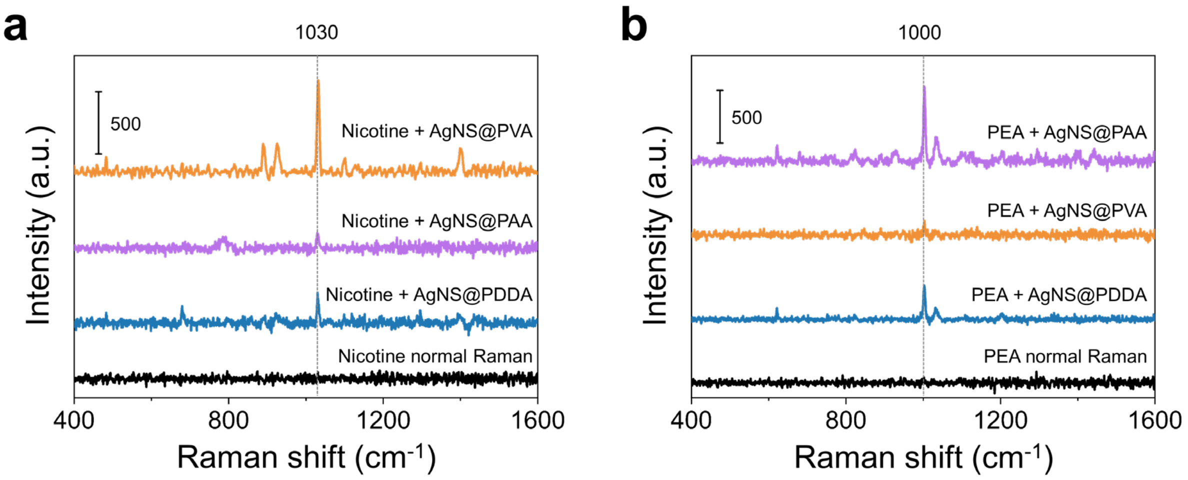

Figure S17. Comparison in SERS Signals of phenethylamine and nicotine with polymer-wrapped nanoprobes. a) SERS signals of 1 mM aqueous nicotine were measured after respectively mixed with AgNS@PVA (orange), AgNS@PAA (purple), AgNS@PDDA (dark blue) and compared with normal Raman spectrum for 1 mM of aqueous nicotine (black). b) SERS signals of 10 mM aqueous phenethylamine were measured after respectively mixed with AgNS@PAA (purple), AgNS@PVA (orange), AgNS@PDDA (dark blue) and compared with normal Raman spectrum for 10 mM of aqueous phenethylamine (black).

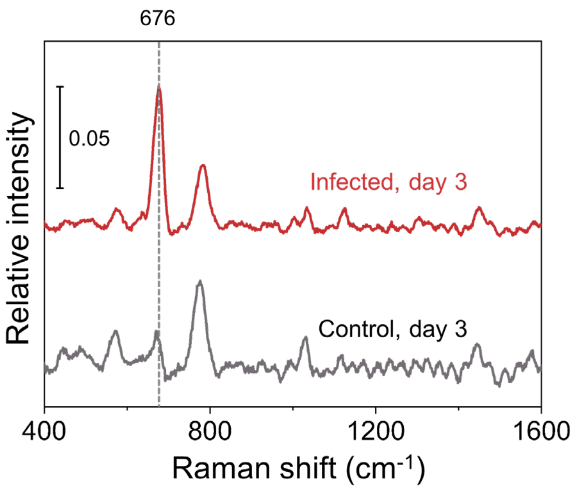

Figure S18. Representative SERS spectrum of volatiles from gray mold-infected strawberry fruit (red) and from control strawberry fruit without infection (gray) on day 3.

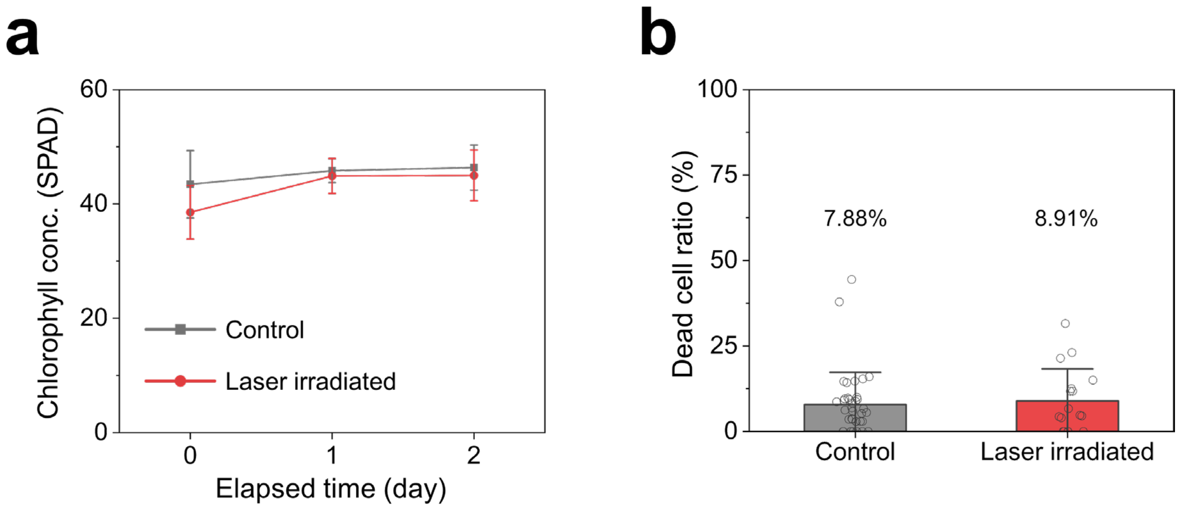

Figure S19. a-b) Chlorophyll content (a) and dead cell ratios of propidium iodide-stained leaf epidermal cell walls (b) of clover plant leaves after nanosensor infiltration and irradiation with 785 nm laser lgiht of 2 mW.

Table S1. Stomata conductance according to stomatal aperture size.

Stomatal aperture, f (μm)	3.4	1.2	0.4
Stomatal conductance, g_s (m/s)	7.87×10^{-4}	3.42×10^{-4}	1.40×10^{-4}

Table S2. Analysis of stomata aperture size and distance between stomata on leaves of clover and watercress.

Plant specimen	Stomatal size (μm)	Distance (μm)
Clover (N=3)	3.4±0.4	87.8±18.5
Watercress (N=3)	3.5±0.3	92.2±20.5

References

[1] C. Willmer, M. Fricker, *Stomata*, Vol. 2, Springer Science & Business Media, **1996**.
[2] B. E. Poling, *The properties of gases and liquids*, **2004**.
[3] A. S. Sharipov, B. I. Loukhovitski, C.-J. Tsai, A. M. Starik, *Eur. Phys. J. D* **2014**, 68, 1.
[4] J. Benitez, *Principles and modern applications of mass transfer operations*, John Wiley & Sons, **2016**.
[5] J.-Y. Parlange, P. E. Waggoner, *Plant Physiol.* **1970**, 46, 337.
[6] T. Vesala, *J. Theor. Biol.* **1998**, 194, 91.
[7] P. C. Harley, in *Biology, controls and models of tree volatile organic compound emissions*, Springer, 2013.
